# Identifying management opportunities to combat climate, land, and marine threats across less climate exposed coral reefs

**DOI:** 10.1111/cobi.13856

**Published:** 2021-12-14

**Authors:** Caitlin D. Kuempel, Vivitskaia J. D. Tulloch, Alyssa L. Giffin, B. Alexander Simmons, Valerie Hagger, Carol Phua, Ove Hoegh‐Guldberg

**Affiliations:** ^1^ Australian Research Council Centre of Excellence for Coral Reef Studies University of Queensland St. Lucia Queensland Australia; ^2^ School of Biological Sciences University of Queensland St. Lucia Queensland Australia; ^3^ Centre for Biodiversity and Conservation Science University of Queensland St. Lucia Queensland Australia; ^4^ Conservation Decisions Lab, Department of Forest and Conservation Science University of British Columbia Vancouver British Columbia Canada; ^5^ Coastal and Marine Research Centre, Australian Rivers Institute – Coast & Estuaries, and School of Environment and Science Griffith University Gold Coast Queensland Australia; ^6^ Global Development Policy Center Boston University Boston Massachusetts USA; ^7^ Institute for Future Environments Queensland University of Technology Brisbane Queensland Australia; ^8^ WWF Oceans Practice Brisbane Queensland Australia

**Keywords:** anthropogenic pressures, climate refugia, conservation investment, cumulative impacts, ecosystem‐based management, threat maps, impactos acumulativos, inversión en la conservación, manejo con base en el ecosistema, mapas de amenazas, presiones antropogénicas, refugios climáticos

## Abstract

Conserving coral reefs is critical for maintaining marine biodiversity, protecting coastlines, and supporting livelihoods in many coastal communities. Climate change threatens coral reefs globally, but researchers have identified a portfolio of coral reefs (bioclimatic units [BCUs]) that are relatively less exposed to climate impacts and strongly connected to other coral reef systems. These reefs provide a proactive opportunity to secure a long‐term future for coral reefs under climate change. To help guide local management efforts, we quantified marine cumulative human impact (CHI) from climate, marine, and land pressures (2013 and from 2008 to 2013) in BCUs and across countries tasked with BCU management. Additionally, we created a management index based on common management measures and policies for each pressure source (climate, marine, and land) to identify a country's intent and commitment to effectively manage these pressures. Twenty‐two countries (79%) had increases in CHI from 2008 to 2013. Climate change pressures had the highest proportional contribution to CHI across all reefs and in all but one country (Singapore), but 18 BCUs (35%) and nine countries containing BCUs (32%) had relatively high land and marine impacts. There was a significant positive relationship between climate impact and the climate management index across countries (*R* ^2^ = 0.43, *p* = 0.02), potentially signifying that countries with greater climate impacts are more committed to managing them. However, this trend was driven by climate management intent in Fiji and Bangladesh. Our results can be used to guide future fine‐scale analyses, national policies, and local management decisions, and our management indices reveal areas where management components can be improved. Cost‐effectively managing local pressures (e.g., fishing and nutrients) in BCUs is essential for building a climate‐ready future that benefits coral reefs and people.

## Abstract (es)

Identificación de Oportunidades de Gestión para Combatir las Amenazas Climáticas, Marinas y Terrestres en los Arrecifes de Coral Menos Expuestos al Clima

**Resumen:**

La conservación de los arrecifes de coral es de suma importancia para mantener la biodiversidad marina y para sostener el medio de vida en muchas comunidades costeras. El cambio climático es una amenaza mundial para los arrecifes de coral; aun así, los investigadores han identificado un portafolio de arrecifes de coral (unidades bioclimáticas[UBCs]) que se encuentran relativamente menos expuestos a los impactos climáticos y están conectados a otros sistemas arrecifales. Estos arrecifes proporcionan una oportunidad proactiva de asegurar un futuro a largo plazo para los arrecifes de coral frente al cambio climático. Para ayudar a guiar los esfuerzos locales de manejo, cuantificamos el impacto humano acumulativo (IHA) sobre los ambientes marinos a partir de las presiones climáticas, marinas y terrestres (del 2008 al 2013) en las UBCs y en los países encargados del manejo de estas. Además, creamos un índice de manejo con base en las medidas y políticas comunes de gestión para cada fuente de presión (clima, ambiente marino, suelo) para identificar la intención y el compromiso de cada país para manejar de manera efectiva estas presiones. Veintidós países (79%) tuvieron incrementos en el IHA entre 2008 y 2013. Las presiones por el cambio climático tuvieron la contribución proporcional más alta al IHA en todos los arrecifes y en todos los países excepto uno (Singapur), pero 18 UBCs (35%) y nueve países que cuentan con UBCs (32%) tuvieron impactos terrestres y marinos relativamente altos. Hubo una relación positiva significativa entre el impacto climático y el índice de manejo climático entre los países (*R* ^2^ = 0.43, *p* = 0.02), lo que potencialmente significa que los países con un mayor impacto climático están más comprometidos con su manejo. Sin embargo, esta tendencia estuvo impulsada por las intenciones de manejo climático en Fiyi y en Bangladesh. Nuestros resultados pueden usarse para orientar los análisis a fina escala, las políticas nacionales y las decisiones locales de manejo en el futuro. Nuestros índices de manejo también revelan áreas en donde se pueden mejorar los componentes gestores. El manejo rentable de las presiones locales (p. ej.: la pesca, los nutrientes) dentro de las UBCs es esencial para construir un futuro preparado climáticamente que beneficie a los arrecifes y a las personas.

## INTRODUCTION

Coral reef ecosystems have significant social, ecological, and economic value. They contain over 25% of the world's marine life and generate upward of $29.8 billion net benefit per year (Samonte‐Tan, 2008), and coral reef fisheries are estimated to support ∼6 million people's livelihoods (Cinner, 2014). However, anthropogenic climate change is driving large‐scale transitions of coral reef and associated ecosystems, with an expected loss of 70–90% of reefs by 2050 given 1.5 °C of global warming (IPCC, 2018). Abating or reducing climate change impacts requires global policy coordination and international commitments to reduce greenhouse gas (GHG) emissions, such as adhering to the global surface temperature goals supported by the Intergovernmental Panel on Climate Change (IPCC, 2018). Global inequalities in both GHG emissions and the location of subsequent impacts make this challenging, especially because the highest emitting countries are generally among the least vulnerable to negative effects of climate change (Althor et al., 2016). Spatial decoupling of GHG emissions and coral stress is particularly true for coral reefs in poorer nations and in the western Indian Ocean (Wolff et al., 2015).

The broad range of environmental and ecological conditions across coral reefs make some areas naturally less exposed to climate change impacts than others. Once identified, these low exposure sites present opportunities to reduce local, direct anthropogenic pressures, and protect the future of coral reefs globally (Beyer et al., 2018; Hoegh‐Guldberg et al., 2018). Recent research used clustering algorithms and modern portfolio theory to identify 50 bioclimatic units (BCUs) (i.e., a planning unit containing ∼500 km^2^ of reef area), representing ∼31% of BCUs globally that are least exposed to climate change impacts, have the highest capacity to repopulate reefs in the future, and maximize the likelihood of conservation success in the face of climate change (Beyer et al., 2018). However, the impacts of climate change are just one category of threats facing reefs. For example, overfishing and unsustainable coastal development are generally considered two of the most severe threats to coral reefs by reef experts and managers (Wear 2016). Mitigating local, direct pressures in BCUs is essential for maintaining reef persistence and improving resilience and recovery potential in the future (Anthony et al., 2015; Hoegh‐Guldberg et al., 2018).

Local, direct pressures on coral reefs originate from both the land and sea—from sedimentation and land‐based nutrient pollution to overfishing and invasive species. In cumulative impact mapping, spatial pressure information can be combined with the spatial distribution of habitats or species to quantify pressures (i.e., stressor intensity weighted by habitat vulnerability) across scales. Pressure information across all stressor and habitat combinations can then be normalized and summed to determine the cumulative impact of all given pressures in an area (Halpern et al., 2015, 2008). Previous research has found substantial incongruences exist between pressures and management actions globally (Tulloch et al., 2020). For example, places with high levels of impact from pressures that could be abated by marine protected areas have nearly 60% less marine protected area coverage than low impact areas, making this an urgent area for further research (Kuempel et al., 2019; Stevenson et al., 2020). However, attempts to link threats to potential management intent are still lacking and have yet to be applied to less climate exposed reefs. Such an analysis is particularly timely as nongovernmental and governmental organizations begin to develop management plans in these areas (e.g., WWF Coral Reef Rescue Initiative and Bloomberg Philanthropies Vibrant Oceans Initiative).

Ultimately, environmental pressures and subsequent management responses (or lack thereof) influence the state of coral reefs. We used a global data set on cumulative human impact (CHI) to marine ecosystems (Halpern et al., 2015) to quantify the cumulative impact of climate, land, and marine pressures and the change in pressures from 2008 to 2013 in BCUs and the countries tasked with their management. Further, we created management indices to assess each country's potential intent and commitment to manage impacts in each category. We aimed to identify key disparities between impacts and common management actions and provide information to help allocate conservation resources for further analyses and conservation actions that benefit people and biodiversity.

## METHODS

To guide and strengthen management and policy decisions in countries that have BCUs that are less subject to climate change impacts (as identified in Beyer et al., [2018]) (Table 1), we categorized pressures to BCUs based on their source (climate, land, or marine [Appendix S1]) and quantified the cumulative impact of each category in BCUs and across BCUs within countries. We also developed management indices as proxies for the potential commitment of each country to abate impacts for each category. All analyses were performed using R 3.6 (R Core Team, 2020) and the raster and sf packages (Hijmans, 2020; Pebesma, 2018). All data and code used in our analyses are available from https://github.com/cdkuempel/50_reefs_threats.

**TABLE 1:** Unique bioclimatic unit identifiers (BCUID) of climate‐tolerant reefs in each country

Country	ISO3^a^	BCUID
Australia	AUS	1
French Polynesia	PYF	2
French Polynesia	PYF	3
Australia	AUS	4
Indonesia	IDN	5
Australia	AUS	6
Australia	AUS	7
French Polynesia	PYF	8
Indonesia	IDN	9
Australia	AUS	10
Indonesia	IDN	11
Australia	AUS	12
Indonesia	IDN	13
Malaysia	MYS	13
Singapore	SGP	13
Kenya	KEN	14
Tanzania	TZA	14
Eritrea	ERI	15
Saudi Arabia	SAU	15
Yemen	YEM	15
Philippines	PHL	16
Indonesia	IDN	17
Malaysia	MYS	18
Philippines	PHL	18
Indonesia	IDN	19
Philippines	PHL	20
Djibouti	DJI	21
Eritrea	ERI	21
Somalia	SOM	21
Indonesia	IDN	23
Tanzania	TZA	24
Tanzania	TZA	25
Indonesia	IDN	27
Indonesia	IDN	32
French Polynesia	PYF	33
Philippines	PHL	34
Indonesia	IDN	35
Timor‐Leste	TLS	35
Egypt	EGY	36
Sudan	SDN	36
Indonesia	IDN	38
Kenya	KEN	39
Somalia	SOM	39
Indonesia	IDN	40
Solomon Islands	SLB	42
Indonesia	IDN	43
Bahamas	BHS	44
India	IND	45
Saudi Arabia	SAU	47
India	IND	49
Sri Lanka	LKA	49
Cuba	CUB	50
Fiji	FJI	51
Brazil	BRA	52
Papua New Guinea	PNG	53
Cuba	CUB	54
Dominican Republic	DOM	54
Haiti	HTI	54
Bangladesh	BGD	56
Myanmar (Burma)	MMR	56
Bahamas	BHS	57
Bahamas	BHS	58
Cuba	CUB	58
Cuba	CUB	59
Indonesia	IDN	60
Fiji	FJI	61

^a^ International for Organization for Standardization country code.

### Bioclimatic units

The BCUs least exposed to climate change impacts were identified by Beyer et al. (2018) using modern portfolio theory and 30 metrics across five data categories (thermal history, projected future conditions, cyclones, connectivity, and recent thermal conditions) (Appendix S2) (Beyer et al., 2018). From these data, an aggregate suitability score for each BCU was calculated by averaging across combinations of standardized metrics and expert‐derived weighting of the relative importance of each data category for informing coral reef conservation planning at an approximately 25 km^2^ resolution (Beyer et al., 2018). Several portfolios of BCUs were then identified using an iterative clustering algorithm that maximized the resilience potential of each BCU (based on the 30 metrics) while minimizing the distance from current members of the cluster of cells, with a set rule allowing a maximum of three BCUs in each marine ecoregion. Each BCU was assigned a BCU identification number (BCUID) (Table 1). We considered only portions of BCUs that were within 200 nautical miles of the shore to remove portions within terrestrial boundaries that were artifacts of the data resolution of the BCU analysis. This is also the area where marine conservation actions are most often implemented.

### Cumulative impact data

We used CHI data from Halpern et al. (2015) to calculate the sum of anthropogenic pressures for the global marine area at a 1‐km^2^ resolution in 2013 (rescaled by one period) and the change in cumulative impacts from 2008 to 2013 (rescaled by two periods). The CHI data are based on human pressures, and values are calculated as the product of the intensity of a given stressor in a cell and the vulnerability of each habitat type to that stressor in that cell. In addition to total CHI, we calculated the sum of pressures associated with three primary sources of impacts: climate, land, and marine (Appendix S1). Climate impacts were classified as indirect impacts requiring global action, whereas land and marine impacts were direct impacts that are manageable at the local scale. We determined the percent contribution of each category to total CHI in and across BCUs in each country. To identify temporal trends of these impacts, we also calculated the change in overall CHI and each CHI category from 2008 to 2013 in and across BCUs in each country. Several data sets were excluded from the change in CHI analysis: sea‐level rise, shipping, invasive species, and ocean pollution pressures did not have data across both periods, and ocean acidification, artisanal fishing, and inorganic pollution did not differ between 2008 and 2013 (Appendix S1).

### Management index

To assess the intent and commitment of a country to manage threats from marine, land, and climate impacts, we created a management index for each category across all analyzed countries, similar to Tulloch et al. (2020). Each individual index was calculated as the average of several metrics based on common management approaches for each category (Appendices S3 & S4) that were normalized from 0 to 1 (1, better score; 0, worse score). The climate index included data on the number of ecosystem‐based adaptation (EbA) strategies in each country, following methods from Giffin et al. (2020); extent to which coastal ecosystems are mentioned in accounting, mitigation, or adaptation in country‐level national contributions; and proportional change in carbon dioxide emissions per gross domestic production and per capita from 2005 to 2017 (Crippa et al., 2018). The marine index included data on areal proportion of strict marine protected areas (IUCN I‐IV) in coral reefs in each country's EEZ (IUCN & UNEP‐WCMC, 2019); level of fisheries management effectiveness (Mora et al., 2009); whether the country is a member of the International Coral Reef Initiative; and level of funding for coral reef conservation (UNEP, 2018). The land index included data on the average number of integrated coastal management (ICM) policies across jurisdictions (international, national, and subnational) and areal proportion of strict protected areas (IUCN I‐IV) in each country's land area (IUCN & UNEP‐WCMC, 2019). For a list of metrics considered but subsequently excluded from each index, see Appendix S5.

Each metric component, *X*, was normalized based on the following equation $\left(X-X_{\min}\right)/(X_{\max}-\;X_{\min}$). The final index for each impact category was considered the average of the component normalized scores. When data were not available for a country, the average score across countries in the same UN geopolitical region (e.g., Southern Asia, Eastern Africa, and Melanesia [UN, 1999]) was used, which is a common approach for estimating missing data for global indices (Frazier et al., 2016). Components of each index were tested for collinearity (Appendix S6). In the marine index, there was a significant positive correlation among countries that are members of the International Coral Reef Initiative and fisheries management (*R* ^2^ = 0.44, *p* = 0.02). Both components, however, were maintained with equal weighting because they represented different aspects of marine conservation efforts (one specifically focused on commitment to coral reef management and the other on broader‐scale fisheries management effectiveness) (OECD et al., 2008).

## RESULTS

Results are presented below by BCU and averaged across BCUs in each country. We provide an additional assessment of the pressures in individual BCUs in each country in Appendix S7.

### Coral reef BCU results

**FIGURE 1:**
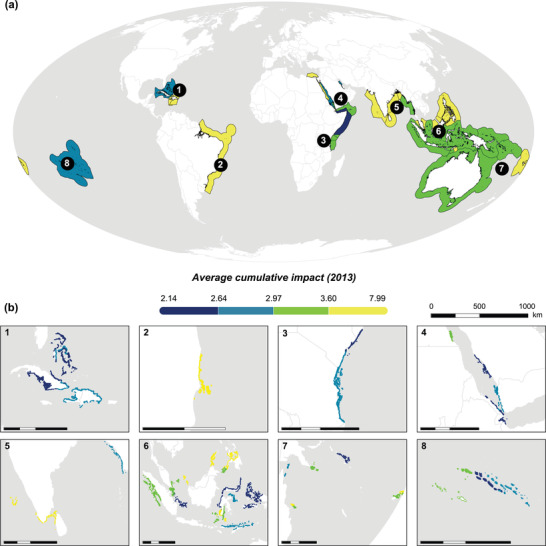
Location and average cumulative impacts for all (a) countries and (b) 50 climate‐resilient bioclimatic units as of 2013. Bioclimatic boundaries thickened to enhance visibility. Color scale represents quantiles. In (a), Exclusive Economic Zones (EEZs) are used for visual purposes; however, actual values were calculated across only the portion of bioclimatic reef units (BCUs) in a country's EEZ, not across the entire EEZ

Across all BCUs, average CHI ranged from 2.3 to 5.8 (mean = 3.5) (Figure 1 & Appendix S8). Eleven BCUs (22%) exhibited relatively high impacts (CHI >4). The BCUs with the highest impacts were in Malaysia, Philippines, and India (BCUID 18, 34, and 45, respectively), whereas BCUs with the lowest impacts were in Cuba and French Polynesia (BCUID 50, 59, and 2, respectively). The majority of BCUs (76%) did not transcend national borders, but eight (16%) and four (8%) BCUs were in two and three countries, respectively (Table 1). No significant correlation was found between a BCU's CHI and the number of countries in that BCU (*R* ^2^ = 0.04, *p* = 0.76).

**FIGURE 2:**
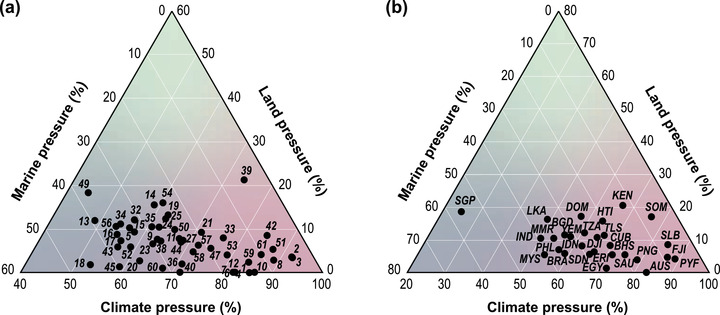
Proportion of cumulative impact from climate, land, and marine pressures in each (a) bioclimatic unit and (b) across bioclimatic units in each country (pink, impacts driven predominantly by climate; blue, impacts driven predominantly by marine pressures; and green, impacts driven predominantly by land pressures). Country abbreviation definitions and bioclimatic unit identifiers are in Table 1

Climate change had the greatest contribution to overall CHI scores across all BCUs, for a mean of 69% (SD 12.4) of cumulative impact (Figure 2). The percentage of impacts attributed to climate change pressures ranged from 44% across BCUs spanning India and Sri Lanka (BCUID 49) to 92% in French Polynesia (BCUID 3). Land‐based marine impacts generally contributed the least to overall CHI; the percent contribution ranged from 0% (BCUIDs = 1, 6, and 7 in Australia) to 21% (BCUID = 39 spanning Kenya and Somalia) (mean = 6.8% [5]). The percentage of marine‐based impacts was slightly higher, ranging from 4.2% (BCUID = 3 in French Polynesia) to 45.4% (BCUID = 18 spanning Malaysia and the Philippines) (mean = 24.3% [10.5]).

**FIGURE 3:**
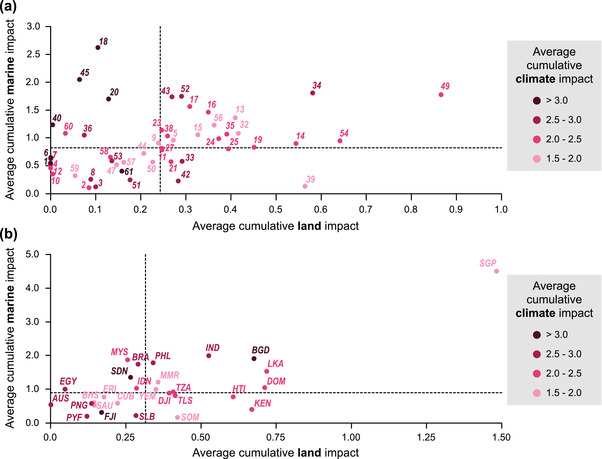
Average marine impact in relation to the average land impact across (a) all 50 bioclimatic units and (b) countries containing bioclimatic units (dashed vertical line, median cumulative marine impact; dashed horizontal line, median cumulative land impact; points to the left of the vertical line, areas with relatively low land impact; points to the right of the vertical line, areas with relatively high impact; points below the horizontal line, areas with relatively low marine impact; and points above the horizontal line, areas with relatively high marine impact). Countries are labeled with their ISO3 codes. Country abbreviation definitions and bioclimatic unit identifiers are in Table 1

Eighteen BCUs (36%) had relatively high land and marine impacts (above or equal to the median impact for each category) (Figure 3a & Appendix S8). Seven BCUs (14%) had relatively high marine impacts and low land impacts. These were located across Indonesia (BCUID = 9, 40, and 60), Malaysia (18), Philippines (18 and 20), India (45), Egypt, and Sudan (36). Similarly, seven BCUs (14%) across 10 countries (BCUID 11, 12, 25, 33, 39, 42, and 30) had relatively high land impacts and low marine impacts. The remaining 18 BCUs (36%) had relatively low land and marine impacts.

**FIGURE 4:**
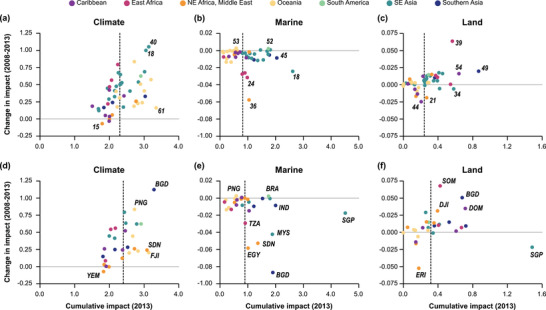
Change in cumulative impact (2008–2013) in relation to cumulative impact in 2013 from climate, marine, and land impacts across 50 climate‐resilient bioclimatic units (a, b, and c, respectively) and countries containing bioclimatic units (d, e, and f, respectively) (dashed vertical line, median cumulative impact; solid horizontal line, 0 change in cumulative impact; points to the left of the vertical line, areas with relatively low impact; points to the right of the vertical line, areas with relatively high impact; points below the horizontal line, decreasing impacts; and points above the horizontal line, increasing impacts from 2008 to 2013). Country abbreviations are defined and bioclimatic unit identifiers are in Table 1

From 2008 to 2013, overall CHI increased in 90% (*n* = 45) of BCUs (Appendix S8). Climate impacts had both the greatest number of BCUs that experienced an increase in impact (94%) and the largest increase in intensity (mean [SD] = 0.37 [0.25]) (Figure 4a). Marine‐based impacts generally decreased across BCUs (mean = −0.07 [0.1]) (Figure 4b); four BCUs had an increase in impact. The BCUs that had the largest increases in CHI were in Indonesia (BCUID = 40, ΔCHI = 1.012), Papua New Guinea (BCUID = 53, ΔCHI = 0.87), Malaysia, and the Philippines (BCUID = 18, ΔCHI = 0.76). Land impacts also increased across 72% of BCUs, but with only a minimal average increase of 0.004 (0.013) (Figure 4c). Decreases in CHI were seen across BCUs in Egypt and Sudan (BCUID = 36, ΔCHI = −0.31), the Bahamas (BCUID = 44, ΔCHI = −0.072), Eritrea, Saudi Arabia, and Yemen (BCUID = 15, ΔCHI = −0.07). Twenty‐four BCUs (48%) had increasing impacts from 2008 to 2013 and relatively high CHI in 2013 (greater than or equal to the median) (Appendices S8 & S9).

### Country‐level results

The BCUs we considered occurred across 28 countries. Nearly one‐third (28%) of countries contained one BCU, whereas Australia and Indonesia contained 6 and 14 BCUs, respectively (Table 1). Total CHI across countries containing BCUs ranged from 2.47 in Somalia to 7.99 in Singapore (mean [SD] = 3.9 [1.18]) (Appendix S10). The relative contribution of climate, land, and marine impacts to CHI remained relatively the same as in BCUs on average (65%, 9.4%, and 25.6%, respectively) (Figure 2b). Singapore had the lowest percentage of CHI attributed to climate change (25%), and French Polynesia had the highest (89%). Conversely, Singapore had the highest proportion of marine CHI (56%), and the Solomon Islands had the lowest (6.7%). Australia had the lowest proportion of land impacts (0.01%) across BCUs, and Kenya had the highest at 20.6%.

Comparing relative marine‐ and land‐based impacts reveals that Djibouti, Haiti, Kenya, Somalia, and Timor‐Leste had relatively higher land‐based impacts than marine impacts across climate‐resilient BCUs, whereas Brazil, Egypt, Indonesia, Malaysia, and Sudan generally had higher marine than land‐based impacts (Figure 3b). Nine countries (Bangladesh, Dominican Republic, India, Sri Lanka, Myanmar, Philippines, Singapore, Tanzania, and Yemen) had relatively high impacts from both land‐ and marine‐based impacts.

Twenty‐two countries (79%) had increases in overall CHI in BCUs, with an average increase in overall CHI of 0.23 (SD 0.28) across all countries (Appendix S10). Papua New Guinea had the largest increase (ΔCHI = 0.87), followed by Timor‐Leste (ΔCHI = 0.72) and Brazil (ΔCHI = 0.66). The Bahamas (ΔCHI = −0.028), Eritrea (ΔCHI = −0.032), Saudi Arabia (ΔCHI = −0.067), Yemen (ΔCHI = −0.072), Sudan (ΔCHI = −0.27), and Egypt (ΔCHI = −0.32) all exhibited decreases during this period.

Climate impacts increased in all but two countries (Saudi Arabia and Yemen), with an average increase of 0.36 (SD 0.28) (Figure 4d). Bangladesh had the largest increase (ΔCHI = 1.12), followed by Papua New Guinea (ΔCHI = 0.83) and Timor‐Leste (ΔCHI = 0.79). Like changes across BCUs, marine impacts decreased by an average of −0.14 (0.21) across countries (Figure 4e). Increases occurred in Papua New Guinea (ΔCHI = 0.027), Brazil (ΔCHI = 0.023), Haiti (ΔCHI = 0.009), and Djibouti (ΔCHI = 0.003). Malaysia, Sudan, Egypt, and Bangladesh all had relatively large decreases in marine impacts (>0.4). Within countries, land‐based impacts experienced relatively little change, with an average increase of 0.009 (0.02) (Figure 4f). Seven countries had decreasing land‐based impacts (Australia, Philippines, Fiji, Bahamas, Saudi Arabia, Singapore, and Eritrea) (Appendix S10). Twelve countries had increasing CHI from 2008 to 2013 and relatively high CHI (greater than or equal to the median) in 2013 (Appendices S9 & S10).

### Management index

We calculated three management indices to estimate the potential intent and commitment of each country to combat each impact category. Higher values indicated a higher potential to manage impacts for each category, whereas lower values indicated a lower potential. Fiji (0.64), Bangladesh (0.62), and Solomon Islands (0.57) had the highest climate management index, whereas Kenya (0.2), Sudan (0.19), and Yemen (0.12) had the lowest (Appendix S11). For the marine index, Tanzania (0.67), Indonesia (0.64), and Australia (0.63) had the highest index, and Djibouti (0.004), Myanmar (0.09), and Saudi Arabia (0.12) had the lowest (Appendix S11). Finally, for the land index, Sri Lanka (0.7), Australia (0.62), and Indonesia (0.62) scored highest, and Solomon Islands (0.07), Timor‐Leste (0.06), and French Polynesia (0) scored lowest (Appendix S11). The land and marine management indices were significantly positively correlated (*R* ^2^ = 0.53, *p* = 0.004).

**FIGURE 5:**
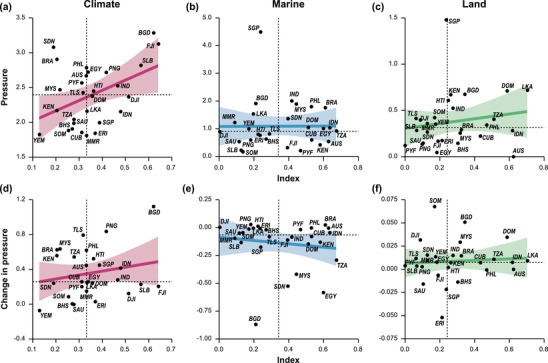
Relationship between (a–c) mean pressure and the management index and (d–f) the change in mean pressure and the management index for each pressure category: (a–d) climate, (b, e) marine, and (c, f) land (vertical dashed lines, median management index; horizontal dashed lines, median pressure value for each category; blue, linear regression trend line; and gray, 95% CIs). Countries in the top left had relatively high pressures but low management indices, countries in the top right had relatively high pressures and high management indices, countries in the bottom left had relatively low pressures and low management indices, and countries in the bottom right had relatively low pressures and high management indices. Countries are labeled by their ISO3 codes. See Table 1 for corresponding country names

The higher the climate management index in a country, the greater the mean climate impact (*R* ^2^ = 0.43, *p* = 0.02) (Figure 5a). The relationship between the marine and land indices and their average pressures were not significant (*R* ^2^ = 0.007, *p* = 0.97 and *R* ^2^ = 0.17, *p* = 0.39, respectively) (Figures 5b & c). Singapore was an outlier for both marine and land impacts, and Fiji and Bangladesh were outliers for the climate index (Appendix S12). When outliers were removed, the relationship between climate pressures and the management index was no longer significant (*R* ^2^ = 0.15, *p* = 0.46), but overall results for land and marine remained the same (*R* ^2^ = 0.3, *p* = 0.13 for land and *R* ^2^ = 0.15, *p* = 0.45 for marine).

Australia, Brazil, Malaysia, Philippines, French Polynesia, Sudan, and Timor‐Leste had relatively high (≥median) climate pressures but low (<median) climate management indices, whereas Bangladesh, Egypt, Fiji, Haiti, India, Papua New Guinea, and Solomon Islands had relatively high impacts and high climate indices. Djibouti, Dominican Republic, Eritrea, Indonesia, Sri Lanka, Myanmar, and Singapore had low climate impacts and high climate indices (Figure 5a).

In the marine realm, Brazil, Dominican Republic, Egypt, Indonesia, India, Malaysia, Philippines, Sudan, and Tanzania had relatively high marine impacts but high marine management indices. Australia, Cuba, Fiji, Kenya, and French Polynesia had relatively low impacts and high marine management indices. Bahamas, Djibouti, Eritrea, Haiti, Papua New Guinea, Saudi Arabia, Solomon Islands, Somalia, and Timor‐Leste had relatively low marine impacts and management indices (Figure 5b). Finally, Bangladesh, Sri Lanka, Myanmar, Singapore, and Yemen had high impacts but low marine management indices.

On land, six countries (Djibouti, Myanmar, Singapore, Somalia, Timor‐Leste, and Yemen) had relatively high land impacts, but low land management indices. Eight countries (Bangladesh, Dominican Republic, Haiti, India, Kenya, Sri Lanka, Philippines, and Tanzania) had relatively high impacts and high land management indices, and six countries (Australia, Bahamas, Brazil, Cuba, Indonesia, and Malaysia) had relatively low impacts but high management indices (Figure 5c). Countries not listed for each realm had low impacts and low management indices.

There was no significant relationship between average change in impacts and the management index for any of the categories (climate: *R* ^2^ = 0.20, *p* = 0.30, marine: *R* ^2^ = −0.14, *p* = 0.48, and land: *R* ^2^ = 0.07, *p* = 0.72). There were seven countries with high change in impacts and a low management index for climate, nine for marine, and seven for land. Conversely, there were seven countries with high change in impacts and a high management index for climate, five for marine, and seven for land (Figures 5d–f).

## DISCUSSION

We quantified cumulative impacts from climate‐, land‐, and marine‐based pressures across coral reef BCUs that have been identified as being relatively less exposed to climate change (thermal stress and cyclones) and more likely to repopulate reefs in the future (Beyer et al., 2018). Additionally, we calculated these threats across BCUs in countries tasked with their management. We linked impacts with policies and management actions to identify a countries’ potential commitment to manage impacts and to help prioritize conservation investments. We found varying contributions from climate‐, marine‐, and land‐based pressures across BCUs and countries. Our approach improves understanding and eases identification of the main source of pressures in BCUs and can be used to document and determine gaps in management measures for specific sources of pressure. When coupled with fine‐scale analysis and ongoing conservation efforts, this approach can help to ensure that BCUs have the best chance of survival.

### Continued need to manage and adapt to climate pressures

Our results highlight the continued need to manage climate pressures at the global scale to secure these areas in the future, as well as the need for a stronger focus on climate adaptation in ecosystems and associated coastal communities. Climate pressures contributed the most to CHI across all BCUs. Although the portfolio of BCUs in our analysis was identified as being relatively less exposed to climate change impacts, tolerance is not synonymous with immunity and there are still many uncertainties surrounding climate impacts, tipping points, and biosphere boundaries (Lenton et al., 2019). Further, these BCUs were identified using data on thermal stress and extreme weather events (Beyer et al., 2018). The climate data we used included different climate pressures (sea surface temperature anomalies, UV radiation anomalies, ocean acidification, and sea‐level rise) that may pose additional risks to these areas. Global action to reduce carbon emissions is still of utmost importance to secure a long‐term future for BCUs and coral reefs globally.

Countries with higher mean climate impacts had higher climate management indices. This suggests that countries experiencing greater impacts of climate change are also those showing the most commitment to tackling it. This trend may, in part, be due to the metrics included in our climate management index and differences in the climate change rhetoric among countries. For example, many organizations that promote and fund EbA projects focus largely on development and adaptation in countries with high climate impacts and low capacity (Doswald et al., 2014; Giffin et al., 2020). Many countries with higher capacity may either continue with traditional marine and land management through a climate change lens without referencing the EbA typology or use more expensive hard infrastructure adaptation solutions (e.g., seawalls) (Giffin et al., 2020; Jones et al., 2012). Finally, many countries with strong climate commitments also rely on substantial external funding, so actual achievement of their targets and commitments may be more uncertain. It appears that a few countries are driving this trend, specifically high levels of climate change management commitment in Fiji and Bangladesh, because this relationship was no longer significant when these countries were removed from the analysis.

Ensuring positive action results from EbA and climate policies (e.g., nationally determined contributions) will be necessary to ensure change, but the commitment of countries experiencing climate change impacts in tackling these threats is promising. Climate change is a global problem and greater investment and collective action from countries not just experiencing climate impacts but contributing most to climate change is needed to alleviate climate pressures across coral reefs globally (Wolff et al., 2015). Further, coral reef resilience to climate impacts could be enhanced by reducing other locally manageable marine and land stressors (Anthony et al., 2015; Brown et al., 2013; Delevaux et al., 2018) or through innovative, yet controversial approaches, such as creating more stress‐tolerant coral assemblages (van Oppen et al., 2015).

### Reducing localized pressures on land and sea

The BCUs and countries with dominant land or marine‐based impacts (upper left and lower right quadrant Figure 3) can achieve the greatest reductions in CHI by focusing efforts on these respective realms. The areas with both high land and marine impacts will require a much more integrated approach to reduce threats across realms. These areas may be particularly at risk due to exposure to multiple sources of impacts, such as potential synergistic interactions among fishing, land‐based run‐off, and climate change pressures. Further, 24 BCUs exhibited relatively high CHI in 2013 and increases in CHI since 2008 (Appendices S8 & S9). These BCUs may be areas in need of urgent conservation action. For example, of these BCUs, 19 had increases in locally manageable land or marine impacts that could potentially be mitigated. Exploring BCUs and countries that have relatively low impacts or relatively small increases in impacts may reveal effective policies, practices, and opportunities to learn. However, there was no significant relationship between marine and land impacts or changes in impacts and their management indices.

The lack of significance between marine and land management indices and pressures could be the result of a lack of commitment or opportunity for countries with high impacts to implement management policies and measures; ineffective conservation actions at reducing these pressures; inadequate or missing components in deriving our management indices; or all three. In the first case, countries with high pressures and low management indices across impact categories (Figure 5) may indicate a need to further develop adaptation and management strategies across these realms. However, it is likely that marine and land management metrics do not adequately reflect outcomes, and compared with our climate management index, the marine and land management metrics lacked measures of concrete change in the drivers of pressures (e.g., change in GHG emissions) due to data gaps or unavailability.

### Prioritizing conservation actions

We found heterogeneous contributions of land and marine impacts across BCUs and countries, which can help guide management decisions. In areas with a high proportion of land‐based impacts, reducing land‐based run‐off from agriculture and waste management (e.g., pesticides, inorganic pollution, and fertilizer pollution [Anthony et al., 2015; Brown et al., 2013]) and improved planning for development projects to reduce impacts (i.e., avoid, minimize, restore, and offset mitigation hierarchy [Arlidge et al., 2018; Kiesecker et al., 2010]) will be necessary to provide the best conservation benefits. Kenya is one such example from our analysis, where land impacts are high. Encouragingly, Kenya already has several ICM strategies in place (two multinational, four national, and one subnational). Explicitly incorporating subnational policies and targeting these efforts to consider climate tolerant reefs would help reduce overall impacts and perhaps increase reef resilience to other stressors (Anthony et al., 2015; Brown et al., 2013).

In the case of marine impacts, climate‐tolerant BCUs in Singapore had the highest proportion of marine‐based impacts across all countries. Exploring the data further revealed high average stressor intensity across four marine stressors: ocean pollution, invasive species, shipping, and night lights, with relatively high habitat vulnerability to invasive species. Given the link between shipping and invasive species (Lim & Tan, 2018), stricter national policies that reduce the introduction of species through ballast water could help reduce these threats (e.g., prioritizing high‐risk vessels and high‐risk nonindigenous marine species [Lim et al., 2017]), but currently they do not exist. Fine‐scale analyses and exploring how nuances in individual pressures and habitat vulnerability scores drive cumulative impact scores can provide more concrete policy recommendations to manage specific threats and should be explored further.

### Comparing BCU and country‐level results

Examining BCUs at the individual and country levels can help prioritize actions across these relatively large BCU areas (500 km^2^) and inform interventions across different levels (e.g., national level policy vs. on‐the‐ground action in BCUs). For example, in Kenya and Somalia, land‐based impacts are greater than marine‐based impacts across the country, suggesting that best‐practice land‐use policies (e.g., ICM and ridge‐to‐reef planning) should be implemented at the national level. However, both countries included two BCUs, and at the BCU level, one BCU had higher marine impacts and one had higher land impacts, suggesting potentially vastly different management approaches in each BCU.

Notably, BCUs with higher marine impacts were shared across multiple countries (BCUID 14 across Kenya and Tanzania and BCUID 21 across Somalia, Eritrea, and Djibouti). This trend was also seen in Singapore, which had one identified BCU that also spanned parts of Indonesia and Malaysia (BCUID = 13). The BCU had a CHI of 3.45, but the portion in Singapore's borders had much higher average impacts (CHI = 7.99), driven by high levels of marine‐based impacts. These nuances are important to consider, and finer‐scale analyses should be conducted in the future to uncover further patterns and dominant pressures driving these trends.

### Limitations and future directions

Although we aimed to use the best global data available to assess cumulative impacts and management potential, several caveats are important to consider. First, indices provide quick measures and benchmarks of the state of conservation actions between areas and through time but are notoriously challenging to construct. It is challenging to find causality between pressures and conservation actions through indices (Moriarty et al., 2018). However, we believe that our indices provide useful information for comparisons among countries, and their individual components provide a better understanding of the state of management actions and policies across climate, marine, and land impacts. Our management indices serve as a proxy for the potential to manage impacts from each pressure category but do not measure effectiveness of these management measures. Determining whether lower impacts are the result of the absence of pressures or the adequate use of policy and management practices to limit these pressures could help implement effective sustainability measures in other areas. Future work should undertake conservation impact and counterfactual analyses of policies and conservation actions at local scales to better understand the degree to which they influence cumulative pressures to help improve these indices.

Second, there are likely important pressures and management metrics we did not consider that may vary by country and BCU. Tourism, mining, and wastewater, for example, are likely to affect several of these BCUs (Burke et al., 2011) but were not accounted for in our analysis due to a lack of global data on these pressures. Seven of the 19 pressures were not accounted for in the change in CHI analyses, as in Halpern et al. (2015), which likely led to underestimates of change. We assumed an equal contribution of each pressure to CHI and included all pressures and habitat types (not only coral reefs) used in Halpern et al. (2015). We included all pressures because of a lack of understanding of how pressures may potentially interact and their relative importance across areas. We included all habitat types due to the large size of BCUs that contained other marine habitats outside of coral reefs, intrinsic linkages between marine habitats, and the need for ecosystem‐based management. We recognize that several pressures may have weaker links to coral reef systems (e.g., pelagic bycatch [Appendices S1 & S13]) and that pressure‐specific weightings or a focus solely on coral reefs would affect our results (Moriarty et al., 2018). Similarly, our management indices may be missing key metrics that we were unable to include due to data gaps or data unavailability. Some of our metrics may have variability within countries that may lead to over or underestimation. For example, our marine management index had a strong focus on fisheries management, and we did not include ICM plans with multipurpose objectives for climate change adaptation, which may underestimate land‐based marine pressures in countries that combine these goals. Similarly, other effective conservation measures, management effectiveness, and community involvement/support likely play large roles in conservation outcomes, but data were either unavailable or did not provide appropriate coverage across our countries of interest (Appendix S5).

Finally, our analysis uncovered broad‐scale patterns in each region and BCU, but additional fine‐scale analysis should be undertaken to ensure the most effective management decisions are made. In doing so, additional aspects of climate vulnerability could be explored, such as adaptive capacity and sensitivity (Cinner et al., 2012; Hughes et al., 2012), and localized habitat vulnerability and pressure scores could be used to further refine cumulative impact assessments.

Careful monitoring and management of coral reefs that are least exposed to climate change is essential for ensuring the greatest chances of their survival and ability to repopulate other areas. Exploring the contribution of climate, land, and marine pressures to CHIs helps prioritize conservation and management efforts to reduce the most prominent local pressures to these BCUs, thereby reducing the overall human impact and increasing resilience. Country‐level patterns can be used to guide national policies, and nuances in individual BCUs are important for guiding local management decisions. Our management indices revealed areas where management components can be improved. Increased commitment to climate change policy, fine‐scale analyses to further inform on‐the‐ground decision‐making, and additional consideration of potential synergies and trade‐offs among pressures and overall conservation outcomes are urgently required to protect these invaluable ecosystems.

## Supporting information

Additional supporting information may be found in the online version of the article at the publisher's website.
**Appendix S1**. Pressures categorized by sources (climate, land, and marine) considered in our analysis. Pressures in bold* were only accounted for in the 2013 analysis but did not have data, or data did not differ, across both time periods so were excluded from the change in pressure analysis (2008–2013).
**Appendix S2**. List of metrics used to identify climate resilient reefs from Beyer et al. (2018).
**Appendix S3**. A list of the management metrics included in each management index (climate, marine, and land), their data source, rationale for inclusion , and key assumptions. See Appendix S4 for detailed methods and assumptions.
**Appendix S4**. Rationale, methodology, and assumptions/limitations for each metric included in the management indices (climate, marine, and land)
**Appendix S5**. Additional metrics that were considered for each management index and the rationale for excluding them from the final metric.
**Appendix S6**. Correlation matrices for components of each conservation index for (a) climate, (b) marine, and (c) land.
**Appendix S7**. Cumulative impact and change in cumulative impact (2008–2013) results by driver across individual BCUs within countries.
**Appendix S8**. Cumulative impact and change in cumulative impact (2008–2013) results by category across bioclimatic units.
**Appendix S9**. The relationship between average total change in cumulative human impact and cumulative human impact in 2013 across (a) coral reef bioclimatic units and (b) countries containing bioclimatic units.
**Appendix S10**. Cumulative impact and change in cumulative impact (2008–2013) results by category across countries containing bioclimatic units
**Appendix S11**. Conservation index for each pressure category by country
**Appendix S12**. Outlier countries for (a) mean pressure and (b) conservation index for each pressure category.
**Appendix S13**. Data distribution of the individual pressures of the cumulative human impact metric by pressures driver category.
