# Supplementary material for: Identifying management opportunities to combat climate, land, and marine threats across less climate exposed coral reefs
Source: Conserv Biol. 2021 Dec 14;36(3):e13856. doi: 10.1111/cobi.13856 (PMC9300080; doi:10.1111/cobi.13856)
Supplement: Supplementary file 1 — Additional supporting information may be found in the online version of the article at the publisher's website. Appendix S1. Pressures categorized by sources (climate, land, and marine) considered in our analysis. Pressures in bold* were only accounted for in the 2013 analysis but did not have data, or data did not differ, across both time periods so were excluded from the change in pressure analysis (2008–2013). Appendix S2. List of metrics used to identify climate resilient reefs from Beyer et al. (2018). Appendix S3. A list of the management metrics included in each management index (climate, marine, and land), their data source, rationale for inclusion , and key assumptions. See Appendix S4 for detailed methods and assumptions. Appendix S4. Rationale, methodology, and assumptions/limitations for each metric included in the management indices (climate, marine, and land) Appendix S5. Additional metrics that were considered for each management index and the rationale for excluding them from the final metric. Appendix S6. Correlation matrices for components of each conservation index for (a) climate, (b) marine, and (c) land. Appendix S7. Cumulative impact and change in cumulative impact (2008–2013) results by driver across individual BCUs within countries. Appendix S8. Cumulative impact and change in cumulative impact (2008–2013) results by category across bioclimatic units. Appendix S9. The relationship between average total change in cumulative human impact and cumulative human impact in 2013 across (a) coral reef bioclimatic units and (b) countries containing bioclimatic units. Appendix S10. Cumulative impact and change in cumulative impact (2008–2013) results by category across countries containing bioclimatic units Appendix S11. Conservation index for each pressure category by country Appendix S12. Outlier countries for (a) mean pressure and (b) conservation index for each pressure category. Appendix S13. Data distribution of the individual pressures of the cu [file COBI-36-0-s001.docx]

**­­Identifying management opportunities to combat climate, land, and marine threats across less climate exposed coral reefs**

**Appendices**

­

**Appendix 1.** Pressures categorized by sources (climate, land, marine) considered in our analysis. Pressures in bold* were only accounted for in the 2013 analysis but did not have data, or data did not differ, across both time periods so were excluded from the change in pressure analysis (2008-2013).

**Appendix 2.** List of metrics used to identify climate resilient reefs from Beyer et al. (2018).

**Appendix 3.** A list of the management metrics included in each management index (climate, marine, land), their data source, rationale for inclusion, and key assumptions. See Appendix 4 for detailed methods and assumptions.

**Appendix 4.** Rationale, methodology, and assumptions/limitations for each metric included in the management indices (climate, marine, land)

**Appendix 5.** Additional metrics that were considered for each management index and the rationale for excluding them from the final metric.

**Appendix 6.** Correlation matrices for components of each conservation index for A) climate, B) marine and C) land.

**Appendix 7.** Cumulative impact and change in cumulative impact (2008 to 2013) results by driver across individual BCUs within countries.

**Appendix 8.** Cumulative impact and change in cumulative impact (2008 to 2013) results by category across bioclimatic units.

**Appendix 9.** The relationship between average total change in cumulative human impact and cumulative human impact in 2013 across A) coral reef bioclimatic units and B) countries containing bioclimatic units.

**Appendix 10.** Cumulative impact and change in cumulative impact (2008 to 2013) results by category across countries containing bioclimatic units

**Appendix 11.** Conservation index for each pressure category by country

**Appendix 12.** Outlier countries for A) mean pressure and B) conservation index for each pressure category.

**Appendix 13.** Data distribution of the individual pressures of the cumulative human impact metric by pressures driver category.

**Appendix 1.** Pressures categorized by sources (climate, land, marine) considered in our analysis. Pressures in bold* were only accounted for in the 2013 analysis but did not have data, or data did not differ, across both time periods so were excluded from the change in pressure analysis (2008-2013).

| **Source** | **Pressures** | **Data Source** | **Rationale** |
| --- | --- | --- | --- |
| Climate | Sea surface temperature anomalies | Coral Reef Temperature Anomaly Database (CoRTAD version 4) | Thermal stress increases the risk of mass coral bleaching events (Heron et al. 2016; Hughes et al. 2017) |
|  | Ultraviolet (UV) radiation anomalies | Number of positive monthly anamolies during a 5-year time from of Local Noon Erythemal UV Irradiance from Aura/OMI satellite data disc.sci.gsfc.nasa.gov/data-holdings/PIP/erythemal_uv_irradiance.shtml | Changes in UV radiation can cause death, growth inhibition and bleaching on coral reefs, ultimately impact coral reef community structure (Shick et al. 1996) |
|  | **Ocean acidification*** | Difference in aragonite saturation state of the ocean circa 1870 and 2000-2009 (Guinotte et al. 2003) | Changes in aragonite saturation state reduce the calcifying ability of corals (Chan & Connolly 2013) |
|  | **Sea level rise*** | Rate of sea level rise (mm/yr) from Nicholls and Cazenave (http://www.aviso.altimetry.fr/en/data/products/ocean-indicatorsproducts/mean-sea-level/products-images.html) | High rates of sea level rise can outpace coral reef growth, effectively drowning corals, and can cause greater land erosion (Perry et al. 2018). |
| Land | Population density | Sum of coastal human population within 10 km radius from the coast using LandScan 30 arc-second population data (http://www.ornl.gov/sci/landscan/) | Population density serves as a proxy for coastal engineering, intertidal trampling, and noise pollution from land. |
|  | Nutrients | Annual FAO country-level fertilizer use | Fertilizer pollution increases nutrients on reefs, which can increase growth rates of competing algae, create dead zones, and increase coral susceptibility to disease and bleaching (Vega Thurber et al. 2014; Wurtsbaugh et al. 2019). |
|  | Light pollution | DMSP stable night lights data layers obtained from NOAA NGDC (<http://ngdc.noaa.gov/eog/dmsp/downloadV4composites.html>), masking out marine areas | Light pollution can disrupt the reproductive cycle of coral reefs and other reef associated species, as well as potentially other physiological, biological, and behavioral cycles (Ayalon et al. 2021). |
|  | Organic pollutants | FAO average annual pesticide (organic pollutants) national statistics | Organic and inorganic pollutions can alter coral calcification, tissue growth, symbiosis, reproduction, and recruitment, ultimately deteriorating coral health (Fabricius 2005) |
|  | **Inorganic pollutants*** | Impervious surface area data (<http://www.ngdc.noaa.gov/dmsp/>) assuming most comes from urban runoff |  |
| Marine | Fishing   - demersal destructive - demersal non-destructive low bycatch - demersal non-destructive high bycatch - pelagic low bycatch - pelagic high bycatch - **artisanal*** | Commercial fishing (demersal and pelagic) was estimated as the percent change in total annual catch per region using catch data per FAO fishing area  Artisanal fishing was based on FAO small-scale fisheries data that was validated by the Sea Around Us Project. | Fishing can have direct effects on coral reefs through habitat destruction and indirect effects through changes in species composition (Jennings & Polunin 1996). Artisanal and demersal fishing are likely the most relevant to coral reefs, but we also include pelagic fishing due to potential unknown effects and interactions between stressors and other coral associated ecosystems. We note pelagic fishing pressure is relatively low compared to other pressures considered in our analysis (Figure S4). |
|  | **Shipping routes*** | Vessel movement paths based on Volunteer Observing System and Automatic Identification System | Shipping can have direct impacts on coral reefs through grounding and habitat destruction and indirect effects through changes in species composition through disturbance (e.g., spatial, noise, dredging) (Burke et al. 2011). |
|  | **Invasive species*** | Volume (tonnes) of goods transported through commercial xfports (World Port Index, National Geospatial- Intelligence Agency) | Invasive species (such as algae, invertebrates and fish) can alter coral reef community structure and reduce abundance, diversity and performance of native coral and fish species (Burke et al. 2011). |
|  | **Ocean pollution*** | Combination of the shipping and port volume (“invasive species”) data layers described above. | Marine-based pollution can undermine coral reef health through oil leaks, ship discharge and solid waste (Burke et al. 2011). |
|  | Benthic structures (oil rigs) | DMSP stable night lights data layers from NOAA NGDC (<http://ngdc.noaa.gov/eog/dmsp/downloadV4composites.html>), masking out land areas | While oil rigs occupy a very small percentage of the ocean, we included this layer as the creation of benthic structures (oil rigs, pipelines) can degrade benthic communities and oil itself can have perverse impacts on coral (and associated ecosystems) survival and reproduction (Burke et al. 2011). Notably, the impact of oil rigs are low in our analysis (Figure S4), and some benthic structures can potentially promote coral growth (e.g., “Rigs to Reefs” (Macreadie et al. 2011). |

**Appendix 2.** List of metrics used to identify climate resilient reefs from Beyer et al. (2018).

| **Metric category** | **Metric** |
| --- | --- |
| **Thermal history (1985–2017)** | Annual sum HotSpots > 0◦C (◦C-days) |
|  | Annual sum HotSpots > 1◦C (◦C-days) |
|  | Annual sum HotSpots > 2◦C (◦C-days) |
|  | Maximum HotSpots, entire record (◦C) |
|  | Annual number of days with HotSpots > 0◦C (days) |
|  | Annual number of days with HotSpots > 1◦C (days) |
|  | Annual number of days with HotSpots > 2◦C (days) |
|  | Maximum DHW (◦C-weeks) |
|  | Annual number of days with DHW > 0 (days) |
|  | Annual number of days with DHW > 4 (days) |
|  | Annual number of days with DHW > 8 (days) |
|  | Trend in annual sum of HotSpots > 0◦C (◦C-days yr−1) |
|  | Trend in summer SST (◦C decade−1) |
| **Projected future conditions (2006-2050)** | Maximum monthly HotSpots, entire record (◦C) |
|  | Maximum DHM, entire record (◦C-months) |
|  | Decadal number of months with HotSpots > 1◦C (months) |
|  | Decadal number of months with HotSpots > 2◦C (months) |
|  | Decadal sum of monthly HotSpots > 1◦C (◦C- months) |
|  | Trend in annual maximum monthly HotSpot (◦C decade−1) |
|  | Trend in annual maximum DHM (◦C-months decade−1) |
|  | Trend in annual sum hotspots > 0◦C (◦C-months decade−1) |
| **Cyclones** | Annual average days exposure (days) |
|  | Annual maximum days exposure (days) |
|  | Inverse return time interval (1 day exposure) (yr−1) |
| **Connectivity** | Larval outgoing settlement (includes self-recruitment; proportion) |
|  | Larval export (no self-recruitment; proportion) |
| **Recent thermal history** | Sum hotspots > 0◦C (degree days yr−1) |
|  | Maximum hotspot (◦C) |
|  | Number days hotspot > 0◦C (d yr−1) |
|  | Maximum DHW (degree weeks) |

**Appendix 3.** A list of the management metrics included in each management index (climate, marine, land), their data source, rationale for inclusion, and key assumptions. See Appendix 4 for detailed methods and assumptions.

| **Impact source** | **Metric** | **Dataset** | **Rationale** | **Method** |
| --- | --- | --- | --- | --- |
| Climate | Number of ecosystem-based adaptation (EbA) strategies in each country | Modified from Giffin et al. (2020) | The more EbA strategies, the more a country could deal with impacts from climate change | Literature review of peer-reviewed and grey literature |
|  | Extent of coastal ecosystems in climate change accounting, mitigation and/or adaptation in Nationally Determined Contributions (NDCs) | (Hagger et al. unpublished data) | The more a country mentions coastal ecosystems in their NDC the more they are committed to combating marine related climate change impacts. | Score of NDC Registry (out of 4) of whether 1) NDC has been submitted, 2) NDC uses IPCC Wetlands Supplement or mentions mangroves or coastal ecosystems in accounting, 3) NDC mentions mangroves or coastal ecosystems in mitigation and 4) NDC mentions mangroves or coastal ecosystems in adaptation |
|  | The proportional change in carbon dioxide emissions per Gross Domestic Product from 2005 to 2017 | Crippa et al. (2018) | The greater the proportional reduction, the more committed a country is to combatting climate change impacts | Values for 2005 and 2017 were taken directly from Crippa et al. (2018), which were calculated based on the Emission Database for Global Atmospheric Research (EDGAR) database. The proportional reduction was then calculated between the two time periods for each country. |
|  | The proportional change in carbon dioxide emissions per capita from 2005 to 2017 | Crippa et al. (2018) | The greater the proportional reduction, the more committed a country is to combatting climate change impacts | Values for 2005 and 2017 were taken directly from Crippa et al. (2018), which were calculated based on the Emission Database for Global Atmospheric Research (EDGAR) database. The proportional reduction was then calculated between the two time periods for each country. |
| Marine | Areal proportion of Marine Protected Areas within coral reefs in each country | IUCN & UNEP-WCMC (2019) | The greater area proportion of MPAs in a country’s EEZ the greater the ability for a country to reduce marine based threats | Strict (IUCN I-IV) marine protected areas intersected with coral reef boundaries (UNEP-WCMC et al. 2018). |
|  | Fisheries management effectiveness | Mora et al. (2009) | The better the fisheries management in a country the better a country is able to manage fishing threats (the number one threat to marine environments globally, and 6/15 marine threat layers across both time periods in the Halpern et al. (2015) dataset). | Values were taken directly from Mora et al. 2009. The study developed a metric for fisheries management sustainability based on a set of survey questions related to the robustness of scientific recommendations, transparency in the translation of science to policy, enforcement capability and compliance with regulations, and extent of fishing capacity, subsidies, and access to foreign fishing. |
|  | Membership/Signatory of the International Coral Reef Initiative | ICRI (2020) | If a country is a member of the International Coral Reef Initiative then they are more committed to safeguarding coral reefs and reducing marine based threats | Binary variable with a 1 signifying membership to the International Coral Reef Initiative |
|  | Coral reef conservation funding | UNEP (2018) | The more money allocated to coral reef conservation in a country, the greater the ability of that country to reduce marine-based threats. | The values were taken directly from UNEP (2018), who reviewed 314 projects from 60 funders. We only considered funding whose end date was later than 2005. |
| Land | Number of Integrated Coastal Management initiatives in each country | Modified methods from Sorensen (2000) | The more integrated coastal management initiatives in a country the greater commitment to reducing land-based marine threats in a country | Literature review conducted to calculate the average number of policies or management plans across scales of management (international, national, and sub-national). |
|  | Areal proportion of terrestrial protected Areas | IUCN & UNEP-WCMC (2019) | The greater area proportion of protected areas in a country, the greater the ability of a country to reduce land-based marine threats in that country | Strict (IUCN I-IV) terrestrial protected areas intersected with country boundaries. |

**Appendix 4.** Rationale, methodology, and assumptions/limitations for each metric included in the management indices (climate, marine, land)

Here, we provide information on the assumptions and methods for each metric included in the three management indices: climate, marine and land (see Table 1 in main text). The management indices were intended to serve as a proxy for a country’s potential ability to combat each impact driver category (climate, marine, land). Each index is a composite of several metrics. Each metric was normalised between 0 and 1 across countries based on the equation $(X-X_{min})/(X_{max}- X_{min}$). The final index for each impact driver was considered as the average across all the normalised component metrics. Additional metrics that were considered for each index, but were subsequently excluded, are listed in Table S4.

Climate metric

*Number of ecosystem-based adaptation (EbA) strategies in each country (modified from Giffin et al. 2020)*

**Rationale:** The more EbA strategies, the more a country could deal with impacts from climate change

**Methodology:** We reviewed both the peer-review and grey literature to identify ecosystem-based adaptation (EbA) 'termed' projects that had been implemented or were in the planning process for each country in the analysis using methodology from Giffin et al. (2020). The peer-review literature was searched in the Scopus database using the term ‘ecosystem-based adaptation’ from 1960 to November 2018 and in the ‘EbA section’ of the Nature-based Solutions Interactive Bibliography database for projects listed before November 2018. To review the EbA grey literature, online EbA databases and published grey literature reports from relevant EbA organisations (i.e., International Union for the Convention of Nature EbA resources, United Nations Framework Convention for Climate Change EbA database, United Nations Environmental Program coastal EbA case studies, Asia Pacific Adaptation Network – EbA adaptation theme, PANORAMA Solutions for a Healthy Planet EbA database, United Nations Development Programme climate change adaptation - EbA and Mitigation and WeAdapt – EbA Adaptation theme) were searched for EbA projects listed before November 2018. Climate change adaptation projects in marine ecosystems identified in a review by Wilson et al. (2020) that met the criteria of EbA as defined in Giffin et al. (2020) were also included.

**Assumptions/Limitations:** We only considered EbA termed projects. Some projects that could be considered relevant may have been missed in the literature search as they were not appropriately termed EbA (e.g., termed disaster risk reduction or nature-based solution instead).

**Gap-filled countries:** None

*Extent of coastal ecosystems in climate change accounting, mitigation and/or adaptation in Nationally Determined Contributions (NDCs)* (Hagger et al. *unpublished data*).

**Rationale:** The more a country mentions coastal ecosystems in their NDC the more they are committed to combating marine related climate change impacts.

**Methodology:** NDCs for mangrove-holding countries from the NDC Registry were reviewed and scored 0-4 based on the following criteria: +1 (NDC submitted, first or second); +1 (uses the IPCC Wetlands Supplement (IPCC 2013) or mentions mangroves or coastal ecosystems in accounting); +1 (mentions mangroves or coastal ecosystems in mitigation); +1 (mentions mangroves or coastal ecosystems in adaptation).

**Assumptions/Limitations:** NDCs reflect a country’s commitment to climate change (mitigation and adaptation) under the Paris Agreement. Most parties have only submitted their Initial NDCs, however Second NDCs are emerging. The last review was undertaken in October 2020 so any commitments in second NCDs submitted after this date have not been captured.

**Gap-filled countries:** None

*The proportional change in carbon dioxide emissions per Gross Domestic Product from 2005 to 2017 (Crippa et al. 2018)*

**Rationale: T**he greater the proportional reduction, the more committed a country is to combatting climate change impacts

**Methodology:** Summarised country-level values of fossil CO_2_ emissions (t CO_2_/1000USD GDP/year) for 2005 and 2017 were taken directly from Crippa et al. (2018) country-level fact sheets in Annex 2. GDP values were taken from the WorldBank (July 2018) expressed in 1000 US dollar and adjusted to the Purchasing Power Parity of 2011. Emission values were calculated based on the Emission Database for Global Atmospheric Research (EDGAR). EDGAR provides spatial and country-level information on anthropogenic greenhouse gas and air pollutant emissions from the past to present. The methodology has four main components:

1. Emissions are calculated using a standardized technology-based (bottom-up) emission factor approach applied across all countries.
2. The calculations apply a consistent set of activity data for calculating emissions comprising the following substances:
   1. Direct greenhouse gases
   2. Ozone precursor gases
   3. Acidfying gases
   4. Primary particulates
   5. Mercury
   6. Stratospheric Ozone Depleting Substances
3. The databse includes a geographic database of emissions at a 0.1 degree by 0.1 degree resolution.
4. Annual and monthly sector-specific emissions timeseries from 1970 to today are also available.

For more information on the EDGAR database and metholodgy see https://edgar.jrc.ec.europa.eu/methodology:

From the 2005 and 2017 values for each country, the proportional reduction in GHG emissions/GDP was then calculated between the two time periods.

**Assumptions/Limitations:** Uncertainty in values is estimated to be ~5% for OECD countries and 10% for non-OECD countries (Crippa et al. 2018). We assume trends remain consistent to today.

**Gap-filled countries:** None

*The proportional change in carbon dioxide emissions per capita from 2005 to 2017 (Crippa et al. 2018)*

**Rationale:** The greater the proportional reduction, the more committed a country is to combatting climate change impacts

**Methodology:** Emissions per capita (tCO_2_/capita/year) for 2005 and 2017 were taken from (Crippa et al. 2018) country-level fact sheets in Annex 2. Emissions were calculated as described above from the Emission Database for Global Atmospheric Research (EDGAR) database. UNDP population statistics data (2017) was used for the population data. The proportional reduction was then calculated between the two time periods for each country.

**Assumptions/Limitations:** Uncertainty in values is estimated to be ~5% for OECD countries and 10% for non-OECD countries (Crippa et al. 2018). We assume trends remain consistent to today.

**Gap-filled countries:** None

Marine metric

*Areal proportion of Marine Protected Areas (MPAs) within coral reefs in each country (IUCN & UNEP-WCMC 2019)*

**Rationale:** The greater area proportion of MPAs in a country’s EEZ the greater the ability for a country to reduce marine based threats

**Methodology:** MPA boundary data was taken from the World Database on Protected Areas (WDPA), the most complete and up-to-date spatial database of protected areas and other effective conservation measures. We cleaned the WDPA database following best practices as in Hanson et al. (2020). The following steps were used to clean the data:

1. The WDPA database was reprojected to an equal-area projection (World Behrman ESRI:54017)
2. UNESCO biosphere reserves and sites with an unknown or proposed designation status were removed
3. Protected areas without delineated boundary data (represented by points) were extracted and buffered according to their reported area using an equidistant coordinate system, and then merged back with the main dataset
4. The dataset was spatially dissolved to remove overlapping areas and slivers were removed to avoid overestimating area

More information and code to reproduce this cleaning methodology is available at <https://github.com/jeffreyhanson/global-protected-areas>

Once the dataset was cleaned, we further subset the data as follows:

1. We only included protected areas designated as “MARINE”
2. We only included areas designated with strict (IUCN I-IV) protection, as these are primarily established for biodiversity protection (Dudley 2008).

Finally, we intersected the cleaned and subset WDPA layer with the WCMC coral reef layer (UNEP-WCMC et al. 2018) to calculate the proportion of coral reefs within strictly marine protected areas within each country.

**Assumptions/Limitations:** MPAs are effectively managed and resourced

**Gap-filled countries:** None

*Fisheries management effectiveness (Mora et al. 2009)*

**Rationale:** The better the fisheries management in a country the better a country is able to manage fishing threats (the number one threat to marine environments globally, and X/13 marine threat layers in the Halpern et al. dataset).

**Methodology:** Values were taken directly from Mora et al. 2009. The study developed a metric for fisheries management sustainability based on a set of survey questions related to the robustness of scientific recommendations, transparency in the translation of science to policy, enforcement capability and compliance with regulations, and extent of fishing capacity, subsidies, and access to foreign fishing. The survey included 23 multiple choice questions in five different languages (English, Spanish, French, Portuguese and German), where participants could rank their answers on a scale from worst- to best-case scenarios. The survey was hosted online and sent via e-mail to 13,892 fishery experts that were collated through a review process of reports on scientific and administrative meetings, webpages of nongovernmental organizations and fishery management organizations and proceedings from international fisheries conferences. Respondants were given one year (April 2007-2008) to complete the survey. In total, the survey received 1,188 positive responses, including at least one reponse from each country with ocean access. Multiple responses for the same zone were averaged. Further tests from Mora et al. (2009) on the survey responses found that they were highly reliable (agreement among responders vs. level of agreement expected from random choice) and valid (high correlation between export opinion and empirical data).

To calculate a single fisheries management effectiveness score for each country, the survey responses were summarized using multidimensional scaling, which is an ordination method that generates hypothetical country responses and uses these as normative extremes to rank real country responses, while incorporating uncertainty through a Monte Caro method.

**Assumptions/Limitations:** Relative fisheries management from 2009 is still similar to that experienced today

**Gap-filled countries:** Singapore, Timor-Leste

*Membership/Signatory of the International Coral Reef Initiative*

**Rationale:** If a country is a member of the International Coral Reef Initiative, they are more committed to safeguarding coral reefs and reducing marine based threats

**Methodology:** Binary variable with a 1 signifying membership to the International Coral Reef Initiative

**Assumptions/Limitations:** Countries maintain/achieve their international commitments, even though they are not legally binding. While this metric is significantly correlated with the fisheries management metric (R^2^ = 0.44, p=0.02), they were both maintained with equal weightings in the final composite metric because we deemed them to be focusing on different and distinct aspects of marine management – one specifically focused on coral reef management and the other on broader scale fisheries management effectiveness. {Citation}

**Gap-filled countries:** None

*Coral reef conservation funding (UNEP 2018)*

**Rationale:** The more money allocated to coral reef conservation in a country, the greater the ability of that country to reduce marine-based threats.

**Methodology:** The values were taken directly UNEP (2018). The UN Environment World Conservation Monitoring Centre conducted a desk-based review between mid-2017 and 2018 and identified 314 projects from 60 funders. Details on the project title, donor, ecosystem, country, region, total cost and co-funding were recorded. They also classified projects within the database under eight broad themes:

1. Conservation and sustainability
2. Marine protected areas management
3. Promoting sustainable living and and alternative livelihoods
4. Fisheries management and governance
5. Climate change resilience
6. Communication and awareness
7. Monitoring and research
8. Policy and legislation

We considered all categories of funding, but only included funding whose end date was later than 2005. The full database can be downloaded from <https://coralfunders.com/>. While the database is the most comprehensive for coral reef funding available, funding data is notoriously difficult to collect and is not always available in the public domain.

**Assumptions/Limitations:** Greater coral reef conservation spending is correlated with greater conservation outcomes

**Gap-filled countries:** Bangladesh, Eritrea, French Polynesia, Singapore

Land metric

*Number of integrated Coastal Management initiatives in each country*

**Rationale:** The more integrated coastal management initiatives in a country the greater commitment to reducing land-based marine threats in a country

**Methodology:** We performed a literature search on Google Scholar search engine including the key search words “coastal management” OR “integrated coastal management” OR “ridge-to-reef planning” OR “land-sea planning” to identify ICM policies and management plans for each country in our analysis. We used the ICM review by Sorensen (2000) to guide initial collation of information. We excluded projects specifically developed for climate change or had “climate” in the title and excluded projects that were primarily marine-based. We calculated the ICM conservation index based on international, national, and sub-national efforts for integrated coastal management by nation. For each scale of management (international, national, and sub-national), we evaluated how many policies or management plans are currently in effect that consider integrated coastal management. We then found the average across the three metrics to obtain a final metric to be normalised across countries.

**Assumptions/Limitations:** Some projects that could be considered relevant may have been missed in the literature search if they were not English-language reports. We assumed published ICM efforts had either been implemented or were in the process of being implemented. We assumed international, national, and sub-national efforts are qual in terms of management application, but, sub-national efforts likely reflect true on-ground management, whilst participation in international agreements may have less relevance to on-ground management.

**Gap-filled countries:** None

*Areal proportion of Protected Areas*

**Rationale:** The greater area proportion of protected areas in a country, the greater the ability of a country to reduce land-based marine threats in that country

**Methodology:** We cleaned the WDPA database following the methods of Hanson et al. (2020), as stated above. For this metric, we only included protected areas designated as “TERRESTRIAL” and with strict (IUCN I-IV) protection, as these are primarily established for biodiversity protection (Dudley 2008). We then intersected the cleaned WDPA layer with boundaries for countries in our analysis to calculate the proportion of terrestrial protected areas within each country.

**Assumptions/Limitations:** We assume protected areas are effectively managed to reduce threats. We recognize that not all terrestrial protected areas may be linked to marine impacts, but believe the metric is still relevant given that many countries we analysed are small island nations and we believe relative differences between countries would remain largely the same if considering coastal protected areas as opposed to all terrestrial protected areas

**Appendix 5.** Additional metrics that were considered for each management index and the rationale for excluding them from the final metric.

| **Impact driver** | **Metric** | **Data source** | **Rationale for exclusion** |
| --- | --- | --- | --- |
| Climate | Signatory to the Paris climate agreement | IPCC (2019) | All are signatories so not meaningful |
|  | ND Gain adaptive capacity | Chen et al. (2015) | Many components are not directly related to marine climate change impacts and adaptive capacity largely refers to adapting to environmental changes not mitigating threats/impact. |
|  | Disaster preparedness score | UN (2015) | Not enough countries with data |
|  | Ease of doing business | World Bank (2020) | Potentially a measure of climate adaptability, but we did not believe it represented a countries commitment or ability to reduce climate pressures |
|  | Coral restoration | Boström-Einarsson et al. (2020) | This metric was excluded for three primary reasons: 1) It is difficult to tell if countries are true zeros or whether the data does not exist or was not collected, 2) the connection between coral restoration and climate mitigation is still unclear, and 3) it is unclear if objective of restoration is truly climate adaptation/resilience |
|  | Monetary value of renewable energy contributions in National Determined Contributions | Cabré et al. (2018) | We would need to assume that nations with higher quantifiable targets are more committed to achieving their NDCs, however 10 countries (34% of countries in analysis) were missing data (Australia and French Polynesia) or did not have quantifiable targets (zero value), thus this assumption did not seem reasonable. |
| Marine | Coral area within each EEZ/total | UNEP-WCMC et al. (2018) | None of the considered threats are directly related to coral reefs so we had no reason to believe the amount of coral area within a country would impact their response to these threats |
|  | % of coral reefs that are less climate exposed (BCUs) | Beyer et al. (2018) and UNEP-WCMC et al. (2018) | BCUs aren’t necessarily widely accepted yet across countries so we do not have reason to believe that the percent of BCUs in a country would impact their commitment to marine management |
|  | Management effectiveness indicators | Gill et al. (2017) | Not enough countries with data |
|  | Signatory to Our Ocean Commitments |  | Significantly positively correlated with the coral reef funding metric (R^2^ = 0.38, p = 0.04). Since commitments are related to actions, which require funding, funding and Our Ocean Commitment components were deemed to represent similar aspects of marine management. In the final composite metric, funding was maintained as it represents a more direct measure of commitment and was not a binary variable. |
| Land | Coastal governance index |  | Not enough countries with data |
|  | Conservation spending | Waldron et al. (2013) | Significantly positively correlated with the ICM metric (R^2^= 0.46, p = 0.01). These two metrics were deemed to represent similar and/or overlapping aspects of land-based management. Further determining the proportion of spending related to land-based *marine* pressures was not possible, thus this component was removed. |

**Appendix 6.** Correlation matrices for components of each conservation index for A) climate, B) marine and C) land.


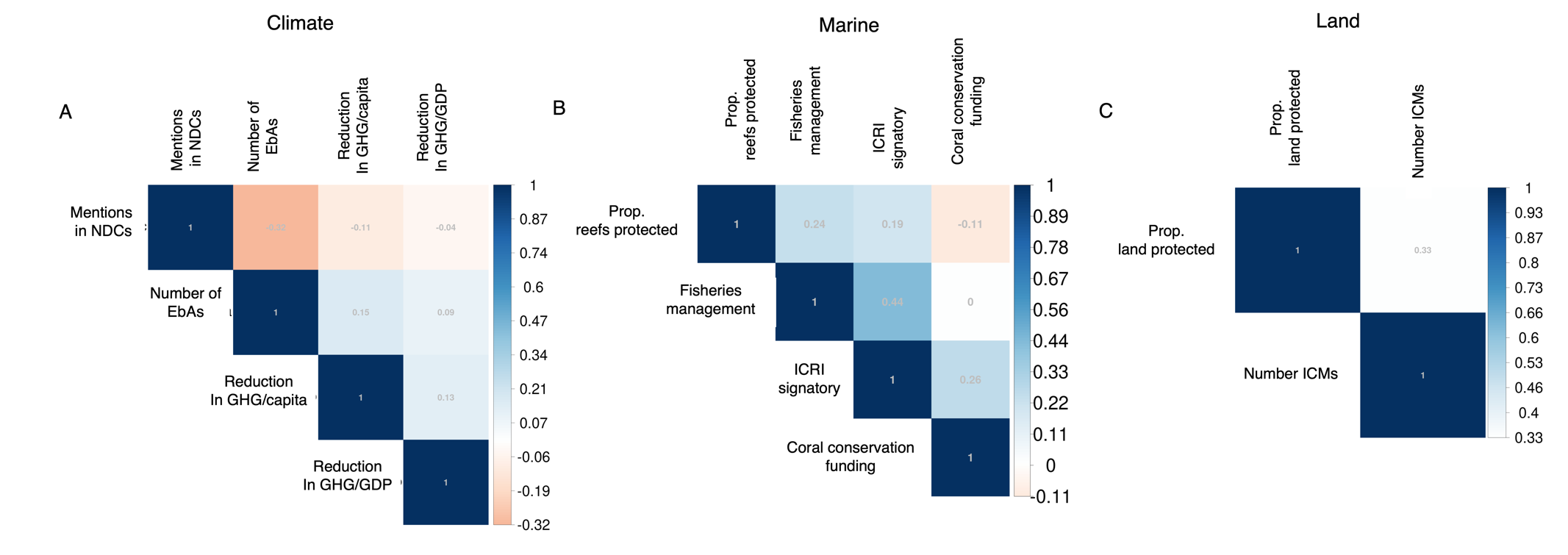


**Appendix 7.** Cumulative impact and change in cumulative impact (2008 to 2013) results by driver across individual BCUs within countries.

| **Country** | **ISO3** | **BCUID** | **Average total cumulative impact (2013)** | **Average cumulative impact by driver (2013)** | | | **Change in average total cumulative impact (2008-2013)** | **Change in average cumulative impact by driver (2008-2013)** | | |
| --- | --- | --- | --- | --- | --- | --- | --- | --- | --- | --- |
|  |  |  |  | **Climate-based** | **Land-based** | **Marine-based** |  | **Climate-based** | **Land-based** | **Marine-based** |
| Australia | AUS | 1 | 3.745 | 3.199 | 0.000 | 0.547 | 0.570 | 0.575 | 0.000 | -0.004 |
| French Polynesia | PYF | 2 | 2.410 | 2.218 | 0.085 | 0.107 | 0.168 | 0.175 | 0.005 | -0.012 |
| French Polynesia | PYF | 3 | 2.925 | 2.703 | 0.100 | 0.122 | 0.165 | 0.186 | 0.003 | -0.024 |
| Australia | AUS | 4 | 2.949 | 2.436 | 0.000 | 0.513 | 0.400 | 0.401 | 0.000 | -0.002 |
| Indonesia | IDN | 5 | 2.928 | 1.696 | 0.272 | 0.960 | 0.072 | 0.122 | 0.013 | -0.063 |
| Australia | AUS | 6 | 3.655 | 3.006 | 0.000 | 0.649 | 0.403 | 0.408 | 0.000 | -0.005 |
| Australia | AUS | 7 | 3.522 | 2.896 | 0.000 | 0.626 | 0.505 | 0.510 | 0.000 | -0.005 |
| French Polynesia | PYF | 8 | 3.119 | 2.772 | 0.089 | 0.258 | 0.256 | 0.261 | 0.005 | -0.010 |
| Indonesia | IDN | 9 | 3.129 | 1.981 | 0.239 | 0.909 | 0.267 | 0.288 | 0.006 | -0.027 |
| Australia | AUS | 10 | 2.656 | 2.296 | 0.005 | 0.356 | 0.308 | 0.308 | 0.001 | -0.001 |
| Indonesia | IDN | 11 | 3.180 | 2.150 | 0.247 | 0.783 | 0.395 | 0.411 | 0.007 | -0.024 |
| Australia | AUS | 12 | 2.648 | 2.187 | 0.000 | 0.461 | 0.395 | 0.402 | -0.001 | -0.005 |
| Indonesia | IDN | 13 | 3.239 | 1.610 | 0.379 | 1.251 | 0.054 | 0.100 | 0.011 | -0.057 |
| Malaysia | MYS | 13 | 3.732 | 1.989 | 0.378 | 1.366 | -0.001 | 0.337 | 0.057 | -0.394 |
| Singapore | SGP | 13 | 7.985 | 1.995 | 1.482 | 4.508 | 0.253 | 0.448 | -0.022 | -0.173 |
| Kenya | KEN | 14 | 3.847 | 2.384 | 0.796 | 0.667 | 0.700 | 0.885 | -0.018 | -0.167 |
| Tanzania | TZA | 14 | 3.374 | 1.935 | 0.449 | 0.990 | 0.160 | 0.448 | 0.008 | -0.296 |
| Eritrea | ERI | 15 | 3.447 | 1.555 | 0.177 | 1.716 | -0.011 | -0.020 | 0.031 | -0.022 |
| Saudi Arabia | SAU | 15 | 2.200 | 1.508 | 0.000 | 0.692 | -0.267 | -0.110 | 0.000 | -0.156 |
| Yemen | YEM | 15 | 3.166 | 1.825 | 0.350 | 0.991 | -0.072 | -0.073 | 0.014 | -0.013 |
| Philippines | PHL | 16 | 4.010 | 2.195 | 0.349 | 1.466 | 0.489 | 0.476 | 0.006 | 0.007 |
| Indonesia | IDN | 17 | 4.275 | 2.403 | 0.309 | 1.564 | 0.076 | 0.171 | 0.013 | -0.107 |
| Malaysia | MYS | 18 | 5.350 | 2.874 | ­­­­­­0.152 | 2.324 | 0.452 | 0.890 | 0.005 | -0.444 |
| Philippines | PHL | 18 | 6.113 | 3.199 | 0.067 | 2.847 | 1.016 | 1.089 | -0.001 | -0.073 |
| Indonesia | IDN | 19 | 3.432 | 2.149 | 0.452 | 0.832 | 0.318 | 0.325 | 0.017 | -0.024 |
| Philippines | PHL | 20 | 4.837 | 3.010 | 0.129 | 1.698 | 0.684 | 0.688 | 0.001 | -0.005 |
| Djibouti | DJI | 21 | 3.651 | 2.366 | 0.394 | 0.891 | 0.156 | 0.122 | 0.032 | 0.003 |
| Eritrea | ERI | 21 | 2.544 | 1.946 | 0.176 | 0.422 | -0.041 | 0.046 | -0.082 | -0.004 |
| Somalia | SOM | 21 | 2.280 | 1.814 | 0.207 | 0.259 | -0.087 | -0.036 | 0.006 | -0.058 |
| Indonesia | IDN | 23 | 3.743 | 2.354 | 0.247 | 1.142 | 0.444 | 0.529 | 0.004 | -0.089 |
| Tanzania | TZA | 24 | 3.598 | 2.242 | 0.373 | 0.983 | 0.493 | 0.796 | 0.014 | -0.316 |
| Tanzania | TZA | 25 | 3.202 | 2.004 | 0.394 | 0.803 | 0.204 | 0.468 | 0.011 | -0.274 |
| Indonesia | IDN | 27 | 3.368 | 2.308 | 0.247 | 0.813 | 0.471 | 0.503 | -0.001 | -0.031 |
| Indonesia | IDN | 32 | 3.442 | 1.944 | 0.417 | 1.081 | 0.408 | 0.449 | -0.006 | -0.035 |
| French Polynesia | PYF | 33 | 3.693 | 2.820 | 0.292 | 0.581 | 0.196 | 0.223 | 0.003 | -0.031 |
| Philippines | PHL | 34 | 5.226 | 2.831 | 0.581 | 1.814 | 0.539 | 0.537 | -0.006 | 0.007 |
| Indonesia | IDN | 35 | 3.724 | 2.238 | 0.388 | 1.097 | 0.640 | 0.661 | 0.005 | -0.026 |
| Timor-Leste | TLS | 35 | 3.648 | 2.427 | 0.414 | 0.807 | 0.722 | 0.793 | 0.012 | -0.083 |
| Egypt | EGY | 36 | 3.777 | 2.721 | 0.048 | 1.008 | -0.317 | 0.258 | 0.007 | -0.583 |
| Sudan | SDN | 36 | 4.710 | 3.081 | 0.266 | 1.364 | -0.268 | 0.242 | 0.015 | -0.526 |
| Indonesia | IDN | 38 | 3.608 | 2.317 | 0.260 | 1.032 | 0.568 | 0.647 | 0.005 | -0.083 |
| Kenya | KEN | 39 | 2.664 | 1.961 | 0.547 | 0.156 | 0.177 | 0.242 | 0.031 | -0.096 |
| Somalia | SOM | 39 | 2.624 | 1.938 | 0.592 | 0.095 | 0.261 | 0.180 | 0.116 | -0.036 |
| Indonesia | IDN | 40 | 4.381 | 3.136 | 0.004 | 1.241 | 1.012 | 1.052 | 0.000 | -0.040 |
| Solomon Islands | SLB | 42 | 3.324 | 2.818 | 0.283 | 0.224 | 0.096 | 0.228 | 0.003 | -0.135 |
| Indonesia | IDN | 43 | 4.600 | 2.590 | 0.269 | 1.741 | 0.373 | 0.405 | 0.009 | -0.042 |
| Bahamas | BHS | 44 | 2.923 | 1.997 | 0.207 | 0.719 | -0.072 | -0.032 | -0.025 | -0.016 |
| India | IND | 45 | 5.137 | 3.027 | 0.064 | 2.046 | 0.247 | 0.330 | 0.002 | -0.086 |
| Saudi Arabia | SAU | 47 | 2.662 | 1.995 | 0.146 | 0.520 | -0.064 | -0.001 | -0.016 | -0.046 |
| India | IND | 49 | 4.963 | 2.042 | 0.968 | 1.953 | 0.177 | 0.233 | 0.027 | -0.082 |
| Sri Lanka | LKA | 49 | 4.412 | 2.160 | 0.718 | 1.534 | 0.254 | 0.248 | 0.009 | -0.004 |
| Cuba | CUB | 50 | 2.300 | 1.508 | 0.227 | 0.565 | 0.123 | 0.188 | 0.013 | -0.078 |
| Fiji | FJI | 51 | 3.370 | 2.947 | 0.177 | 0.247 | 0.141 | 0.238 | -0.004 | -0.094 |
| Brazil | BRA | 52 | 4.943 | 2.906 | 0.290 | 1.747 | 0.663 | 0.625 | 0.014 | 0.023 |
| Papua New Guinea | PNG | 53 | 3.440 | 2.717 | 0.136 | 0.587 | 0.865 | 0.835 | 0.003 | 0.028 |
| Cuba | CUB | 54 | 4.084 | 2.449 | 0.546 | 1.089 | 0.030 | 0.140 | 0.003 | -0.114 |
| Dominican Republic | DOM | 54 | 4.136 | 2.374 | 0.712 | 1.050 | 0.130 | 0.244 | 0.035 | -0.149 |
| Haiti | HTI | 54 | 3.840 | 2.449 | 0.606 | 0.785 | 0.533 | 0.522 | 0.002 | 0.009 |
| Bangladesh | BGD | 56 | 5.869 | 3.284 | 0.675 | 1.910 | 0.304 | 1.123 | 0.051 | -0.869 |
| Myanmar (Burma) | MMR | 56 | 3.382 | 1.809 | 0.356 | 1.217 | 0.065 | 0.149 | 0.010 | -0.094 |
| Bahamas | BHS | 57 | 2.605 | 1.881 | 0.163 | 0.562 | 0.016 | 0.051 | -0.013 | -0.022 |
| Bahamas | BHS | 58 | 2.546 | 1.871 | 0.087 | 0.588 | -0.041 | -0.017 | -0.008 | -0.017 |
| Cuba | CUB | 58 | 3.848 | 2.582 | 0.334 | 0.933 | 0.178 | 0.274 | 0.000 | -0.096 |
| Cuba | CUB | 59 | 2.365 | 1.991 | 0.054 | 0.320 | 0.355 | 0.421 | 0.001 | -0.068 |
| Indonesia | IDN | 60 | 3.451 | 2.334 | 0.033 | 1.084 | 0.481 | 0.509 | 0.003 | -0.031 |
| Fiji | FJI | 61 | 3.905 | 3.345 | 0.158 | 0.402 | 0.021 | 0.163 | -0.009 | -0.133 |

**Appendix 8.** Cumulative impact and change in cumulative impact (2008 to 2013) results by driver across bioclimatic units.

| BCUID | Average total cumulative impact (2013) | Average cumulative impact by driver (2013) | | | Prop. of cumulative impact by driver (2013) | | | Change in average total cumulative impact (2008-2013) | Change in average cumulative impact by driver (2008-2013) | | | Prop. Change in cumulative impact by driver (2008-2013) | | | |
| --- | --- | --- | --- | --- | --- | --- | --- | --- | --- | --- | --- | --- | --- | --- | --- |
|  |  | Climate-based | Land-based | Marine-based | Climate-based | Land-based | Marine-based |  | Climate-based | Land-based | Marine-based | Climate-based | Land-based | Marine-based |  |
| 1 | 3.745 | 3.199 | 0.000 | 0.547 | 0.854 | 0.000 | 0.146 | 0.570 | 0.575 | 0.000 | -0.004 | 0.024 | 0.000 | -0.024 |  |
| 2 | 2.410 | 2.218 | 0.085 | 0.107 | 0.920 | 0.035 | 0.044 | 0.168 | 0.175 | 0.005 | -0.012 | 0.012 | -0.003 | -0.009 |  |
| 3 | 2.925 | 2.703 | 0.100 | 0.122 | 0.924 | 0.034 | 0.042 | 0.165 | 0.186 | 0.003 | -0.024 | 0.014 | -0.002 | -0.012 |  |
| 4 | 2.949 | 2.436 | 0.000 | 0.513 | 0.826 | 0.000 | 0.174 | 0.400 | 0.401 | 0.000 | -0.002 | 0.043 | 0.000 | -0.043 |  |
| 5 | 2.928 | 1.696 | 0.272 | 0.960 | 0.579 | 0.093 | 0.328 | 0.072 | 0.122 | 0.013 | -0.063 | 0.089 | -0.003 | -0.086 |  |
| 6 | 3.655 | 3.006 | 0.000 | 0.649 | 0.822 | 0.000 | 0.178 | 0.403 | 0.408 | 0.000 | -0.005 | 0.023 | 0.000 | -0.023 |  |
| 7 | 3.522 | 2.896 | 0.000 | 0.626 | 0.822 | 0.000 | 0.178 | 0.505 | 0.510 | 0.000 | -0.005 | 0.031 | 0.000 | -0.031 |  |
| 8 | 3.119 | 2.772 | 0.089 | 0.258 | 0.889 | 0.029 | 0.083 | 0.256 | 0.261 | 0.005 | -0.010 | 0.009 | -0.003 | -0.006 |  |
| 9 | 3.129 | 1.981 | 0.239 | 0.909 | 0.633 | 0.076 | 0.290 | 0.267 | 0.288 | 0.006 | -0.027 | 0.106 | -0.018 | -0.088 |  |
| 10 | 2.656 | 2.296 | 0.005 | 0.356 | 0.864 | 0.002 | 0.134 | 0.308 | 0.308 | 0.001 | -0.001 | 0.046 | -0.002 | -0.044 |  |
| 11 | 3.180 | 2.150 | 0.247 | 0.783 | 0.676 | 0.078 | 0.246 | 0.395 | 0.411 | 0.007 | -0.024 | 0.106 | -0.023 | -0.083 |  |
| 12 | 2.648 | 2.187 | 0.000 | 0.461 | 0.826 | 0.000 | 0.174 | 0.395 | 0.402 | -0.001 | -0.005 | 0.132 | -0.002 | -0.130 |  |
| 13 | 3.454 | 1.682 | 0.410 | 1.362 | 0.487 | 0.119 | 0.394 | 0.051 | 0.148 | 0.018 | -0.115 | 0.095 | 0.002 | -0.097 |  |
| 14 | 3.503 | 2.059 | 0.544 | 0.901 | 0.588 | 0.155 | 0.257 | 0.309 | 0.568 | 0.001 | -0.260 | 0.209 | -0.037 | -0.171 |  |
| 15 | 3.174 | 1.793 | 0.327 | 1.055 | 0.565 | 0.103 | 0.332 | -0.070 | -0.069 | 0.015 | -0.017 | -0.019 | 0.018 | 0.002 |  |
| 16 | 4.010 | 2.195 | 0.349 | 1.466 | 0.547 | 0.087 | 0.365 | 0.489 | 0.476 | 0.006 | 0.007 | 0.146 | -0.031 | -0.115 |  |
| 17 | 4.275 | 2.403 | 0.309 | 1.564 | 0.562 | 0.072 | 0.366 | 0.076 | 0.171 | 0.013 | -0.107 | 0.053 | 0.002 | -0.055 |  |
| 18 | 5.779 | 3.052 | 0.105 | 2.622 | 0.528 | 0.018 | 0.454 | 0.759 | 0.999 | 0.002 | -0.242 | 0.168 | -0.004 | -0.164 |  |
| 19 | 3.432 | 2.149 | 0.452 | 0.832 | 0.626 | 0.132 | 0.242 | 0.318 | 0.325 | 0.017 | -0.024 | 0.067 | -0.018 | -0.049 |  |
| 20 | 4.837 | 3.010 | 0.129 | 1.698 | 0.622 | 0.027 | 0.351 | 0.684 | 0.688 | 0.001 | -0.005 | 0.072 | -0.006 | -0.066 |  |
| 21 | 2.920 | 2.082 | 0.268 | 0.571 | 0.713 | 0.092 | 0.195 | 0.027 | 0.058 | -0.019 | -0.013 | 0.026 | -0.016 | -0.010 |  |
| 23 | 3.743 | 2.354 | 0.247 | 1.142 | 0.629 | 0.066 | 0.305 | 0.444 | 0.529 | 0.004 | -0.089 | 0.103 | -0.012 | -0.091 |  |
| 24 | 3.598 | 2.242 | 0.373 | 0.983 | 0.623 | 0.104 | 0.273 | 0.493 | 0.796 | 0.014 | -0.316 | 0.285 | -0.036 | -0.249 |  |
| 25 | 3.202 | 2.004 | 0.394 | 0.803 | 0.626 | 0.123 | 0.251 | 0.204 | 0.468 | 0.011 | -0.274 | 0.225 | -0.020 | -0.205 |  |
| 27 | 3.368 | 2.308 | 0.247 | 0.813 | 0.685 | 0.073 | 0.241 | 0.471 | 0.503 | -0.001 | -0.031 | 0.137 | -0.029 | -0.108 |  |
| 32 | 3.442 | 1.944 | 0.417 | 1.081 | 0.565 | 0.121 | 0.314 | 0.408 | 0.449 | -0.006 | -0.035 | 0.147 | -0.040 | -0.108 |  |
| 33 | 3.693 | 2.820 | 0.292 | 0.581 | 0.764 | 0.079 | 0.157 | 0.196 | 0.223 | 0.003 | -0.031 | 0.021 | -0.006 | -0.015 |  |
| 34 | 5.226 | 2.831 | 0.581 | 1.814 | 0.542 | 0.111 | 0.347 | 0.539 | 0.537 | -0.006 | 0.007 | 0.075 | -0.024 | -0.050 |  |
| 35 | 3.716 | 2.257 | 0.391 | 1.069 | 0.607 | 0.105 | 0.288 | 0.648 | 0.674 | 0.006 | -0.032 | 0.134 | -0.038 | -0.096 |  |
| 36 | 3.891 | 2.765 | 0.075 | 1.052 | 0.711 | 0.019 | 0.270 | -0.311 | 0.256 | 0.008 | -0.576 | 0.170 | 0.006 | -0.177 |  |
| 38 | 3.608 | 2.317 | 0.260 | 1.032 | 0.642 | 0.072 | 0.286 | 0.568 | 0.647 | 0.005 | -0.083 | 0.126 | -0.018 | -0.108 |  |
| 39 | 2.648 | 1.952 | 0.564 | 0.132 | 0.737 | 0.213 | 0.050 | 0.209 | 0.219 | 0.064 | -0.073 | 0.069 | -0.006 | -0.063 |  |
| 40 | 4.381 | 3.136 | 0.004 | 1.241 | 0.716 | 0.001 | 0.283 | 1.012 | 1.052 | 0.000 | -0.040 | 0.171 | -0.001 | -0.170 |  |
| 42 | 3.324 | 2.818 | 0.283 | 0.224 | 0.848 | 0.085 | 0.067 | 0.096 | 0.228 | 0.003 | -0.135 | 0.061 | -0.003 | -0.058 |  |
| 43 | 4.600 | 2.590 | 0.269 | 1.741 | 0.563 | 0.059 | 0.378 | 0.373 | 0.405 | 0.009 | -0.042 | 0.078 | -0.008 | -0.070 |  |
| 44 | 2.923 | 1.997 | 0.207 | 0.719 | 0.683 | 0.071 | 0.246 | -0.072 | -0.032 | -0.025 | -0.016 | 0.016 | -0.009 | -0.008 |  |
| 45 | 5.137 | 3.027 | 0.064 | 2.046 | 0.589 | 0.013 | 0.398 | 0.247 | 0.330 | 0.002 | -0.086 | 0.044 | 0.000 | -0.044 |  |
| 47 | 2.662 | 1.995 | 0.146 | 0.520 | 0.750 | 0.055 | 0.195 | -0.064 | -0.001 | -0.016 | -0.046 | 0.042 | -0.007 | -0.035 |  |
| 49 | 4.736 | 2.090 | 0.866 | 1.780 | 0.441 | 0.183 | 0.376 | 0.209 | 0.239 | 0.020 | -0.050 | 0.060 | -0.013 | -0.047 |  |
| 50 | 2.300 | 1.508 | 0.227 | 0.565 | 0.656 | 0.099 | 0.246 | 0.123 | 0.188 | 0.013 | -0.078 | 0.086 | -0.008 | -0.078 |  |
| 51 | 3.370 | 2.947 | 0.177 | 0.247 | 0.874 | 0.052 | 0.073 | 0.141 | 0.238 | -0.004 | -0.094 | 0.052 | -0.007 | -0.046 |  |
| 52 | 4.943 | 2.906 | 0.290 | 1.747 | 0.588 | 0.059 | 0.353 | 0.663 | 0.625 | 0.014 | 0.023 | 0.076 | -0.019 | -0.056 |  |
| 53 | 3.440 | 2.717 | 0.136 | 0.587 | 0.790 | 0.039 | 0.171 | 0.865 | 0.835 | 0.003 | 0.028 | 0.096 | -0.053 | -0.042 |  |
| 54 | 4.010 | 2.417 | 0.642 | 0.952 | 0.603 | 0.160 | 0.237 | 0.271 | 0.335 | 0.016 | -0.081 | 0.054 | -0.016 | -0.038 |  |
| 56 | 3.431 | 1.838 | 0.362 | 1.231 | 0.536 | 0.106 | 0.359 | 0.070 | 0.169 | 0.010 | -0.109 | 0.069 | -0.001 | -0.068 |  |
| 57 | 2.605 | 1.881 | 0.163 | 0.562 | 0.722 | 0.062 | 0.216 | 0.016 | 0.051 | -0.013 | -0.022 | 0.021 | -0.008 | -0.013 |  |
| 58 | 2.789 | 2.003 | 0.133 | 0.653 | 0.718 | 0.048 | 0.234 | 0.000 | 0.038 | -0.007 | -0.031 | 0.021 | -0.004 | -0.018 |  |
| 59 | 2.365 | 1.991 | 0.054 | 0.320 | 0.842 | 0.023 | 0.135 | 0.355 | 0.421 | 0.001 | -0.068 | 0.074 | -0.007 | -0.067 |  |
| 60 | 3.451 | 2.334 | 0.033 | 1.084 | 0.676 | 0.009 | 0.314 | 0.481 | 0.509 | 0.003 | -0.031 | 0.142 | -0.003 | -0.139 |  |
| 61 | 3.905 | 3.345 | 0.158 | 0.402 | 0.857 | 0.040 | 0.103 | 0.021 | 0.163 | -0.009 | -0.133 | 0.053 | -0.004 | -0.049 |  |

**Appendix 9.** The relationship between average total change in cumulative human impact and cumulative human impact in 2013 across A) coral reef bioclimatic units and B) countries containing bioclimatic units.

**
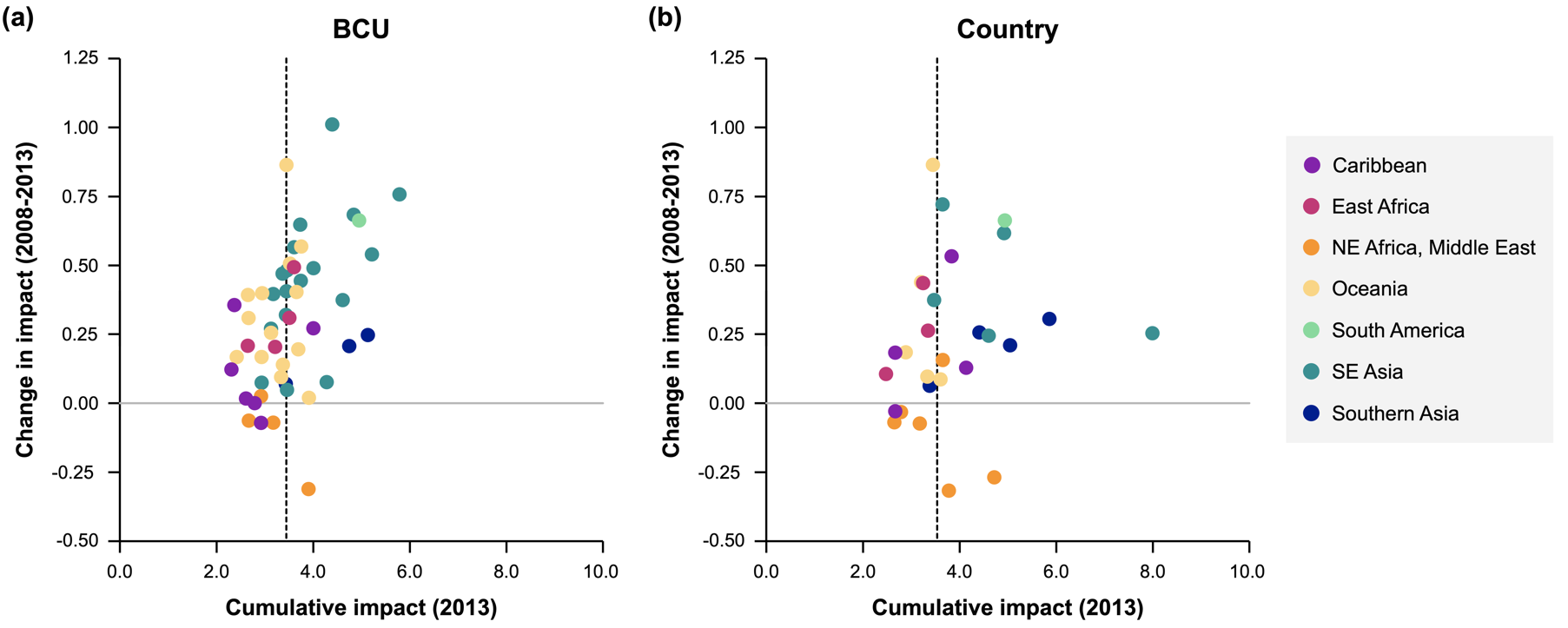
**

**Appendix 10.** Cumulative impact and change in cumulative impact (2008 to 2013) results by driver across countries containing bioclimatic units

| Country | ISO3 | Average total cumulative impact (2013) | Average cumulative impact by driver (2013) | | | Prop. of cumulative impact by driver (2013) | | | Change in average total cumulative impact (2008-2013) | | Change in average cumulative impact by driver (2008-2013) | | | | | | Prop. change in cumulative impact by driver (2008-2013) | | | | | | |
| --- | --- | --- | --- | --- | --- | --- | --- | --- | --- | --- | --- | --- | --- | --- | --- | --- | --- | --- | --- | --- | --- | --- | --- |
|  |  |  | Climate-based | Land-based | Marine-based | Climate-based | Land-based | Marine-based |  |  | Climate-based | | Land-based | | Marine-based | | Climate-based | | Land-based | | Marine-based | |  |
| Australia | AUS | 3.207 | 2.670 | 0.000 | 0.537 | 0.832 | 0.000 | 0.168 | 0.441 | 0.445 | | 0.000 | | -0.004 | | 0.040 | | 0.000 | | -0.040 | |  |  |
| Bangladesh | BGD | 5.869 | 3.284 | 0.675 | 1.910 | 0.560 | 0.115 | 0.325 | 0.304 | 1.123 | | 0.051 | | -0.869 | | 0.201 | | 0.002 | | -0.204 | |  |  |
| Bahamas | BHS | 2.660 | 1.906 | 0.144 | 0.611 | 0.716 | 0.054 | 0.230 | -0.028 | 0.004 | | -0.014 | | -0.018 | | 0.017 | | -0.007 | | -0.010 | |  |  |
| Brazil | BRA | 4.943 | 2.906 | 0.290 | 1.747 | 0.588 | 0.059 | 0.353 | 0.663 | 0.625 | | 0.014 | | 0.023 | | 0.076 | | -0.019 | | -0.056 | |  |  |
| Cuba | CUB | 2.667 | 1.855 | 0.223 | 0.589 | 0.696 | 0.083 | 0.221 | 0.185 | 0.258 | | 0.007 | | -0.081 | | 0.072 | | -0.010 | | -0.061 | |  |  |
| Djibouti | DJI | 3.651 | 2.366 | 0.394 | 0.891 | 0.648 | 0.108 | 0.244 | 0.156 | 0.122 | | 0.032 | | 0.003 | | 0.003 | | 0.002 | | -0.004 | |  |  |
| Dominican Republic | DOM | 4.136 | 2.374 | 0.712 | 1.050 | 0.574 | 0.172 | 0.254 | 0.130 | 0.244 | | 0.035 | | -0.149 | | 0.059 | | 0.001 | | -0.059 | |  |  |
| Egypt | EGY | 3.777 | 2.721 | 0.048 | 1.008 | 0.720 | 0.013 | 0.267 | -0.317 | 0.258 | | 0.007 | | -0.583 | | 0.182 | | 0.005 | | -0.187 | |  |  |
| Eritrea | ERI | 2.779 | 1.842 | 0.177 | 0.761 | 0.663 | 0.064 | 0.274 | -0.032 | 0.029 | | -0.052 | | -0.009 | | 0.043 | | -0.039 | | -0.004 | |  |  |
| Fiji | FJI | 3.609 | 3.125 | 0.168 | 0.316 | 0.866 | 0.047 | 0.088 | 0.087 | 0.205 | | -0.006 | | -0.111 | | 0.052 | | -0.005 | | -0.047 | |  |  |
| Haiti | HTI | 3.840 | 2.449 | 0.606 | 0.785 | 0.638 | 0.158 | 0.204 | 0.533 | 0.522 | | 0.002 | | 0.009 | | 0.055 | | -0.039 | | -0.015 | |  |  |
| Indonesia | IDN | 3.470 | 2.153 | 0.286 | 1.031 | 0.621 | 0.082 | 0.297 | 0.375 | 0.414 | | 0.007 | | -0.046 | | 0.112 | | -0.020 | | -0.092 | |  |  |
| India | IND | 5.048 | 2.524 | 0.526 | 1.998 | 0.500 | 0.104 | 0.396 | 0.211 | 0.280 | | 0.015 | | -0.084 | | 0.051 | | -0.004 | | -0.047 | |  |  |
| Kenya | KEN | 3.246 | 2.169 | 0.669 | 0.407 | 0.668 | 0.206 | 0.125 | 0.434 | 0.558 | | 0.007 | | -0.131 | | 0.170 | | -0.068 | | -0.101 | |  |  |
| Sri Lanka | LKA | 4.412 | 2.160 | 0.718 | 1.534 | 0.490 | 0.163 | 0.348 | 0.254 | 0.248 | | 0.009 | | -0.004 | | 0.059 | | -0.019 | | -0.040 | |  |  |
| Myanmar (Burma) | MMR | 3.382 | 1.809 | 0.356 | 1.217 | 0.535 | 0.105 | 0.360 | 0.065 | 0.149 | | 0.010 | | -0.094 | | 0.062 | | -0.001 | | -0.062 | |  |  |
| Malaysia | MYS | 4.605 | 2.467 | 0.256 | 1.883 | 0.536 | 0.056 | 0.409 | 0.244 | 0.635 | | 0.029 | | -0.421 | | 0.202 | | 0.003 | | -0.205 | |  |  |
| Philippines | PHL | 4.916 | 2.784 | 0.341 | 1.791 | 0.566 | 0.069 | 0.364 | 0.616 | 0.621 | | -0.001 | | -0.004 | | 0.090 | | -0.017 | | -0.073 | |  |  |
| Papua New Guinea | PNG | 3.440 | 2.717 | 0.136 | 0.587 | 0.790 | 0.039 | 0.171 | 0.865 | 0.835 | | 0.003 | | 0.028 | | 0.096 | | -0.053 | | -0.042 | |  |  |
| French Polynesia | PYF | 2.891 | 2.567 | 0.120 | 0.204 | 0.888 | 0.042 | 0.071 | 0.185 | 0.200 | | 0.004 | | -0.019 | | 0.014 | | -0.003 | | -0.011 | |  |  |
| Saudi Arabia | SAU | 2.653 | 1.987 | 0.144 | 0.523 | 0.749 | 0.054 | 0.197 | -0.067 | -0.003 | | -0.016 | | -0.048 | | 0.043 | | -0.006 | | -0.036 | |  |  |
| Sudan | SDN | 4.710 | 3.081 | 0.266 | 1.364 | 0.654 | 0.056 | 0.290 | -0.268 | 0.242 | | 0.015 | | -0.526 | | 0.114 | | 0.010 | | -0.124 | |  |  |
| Singapore | SGP | 7.985 | 1.995 | 1.482 | 4.508 | 0.250 | 0.186 | 0.565 | 0.253 | 0.448 | | -0.022 | | -0.173 | | 0.124 | | -0.042 | | -0.082 | |  |  |
| Solomon Islands | SLB | 3.324 | 2.818 | 0.283 | 0.224 | 0.848 | 0.085 | 0.067 | 0.096 | 0.228 | | 0.003 | | -0.135 | | 0.061 | | -0.003 | | -0.058 | |  |  |
| Somalia | SOM | 2.472 | 1.883 | 0.422 | 0.167 | 0.762 | 0.171 | 0.068 | 0.107 | 0.085 | | 0.068 | | -0.046 | | 0.021 | | 0.028 | | -0.048 | |  |  |
| Timor-Leste | TLS | 3.648 | 2.427 | 0.414 | 0.807 | 0.665 | 0.114 | 0.221 | 0.722 | 0.793 | | 0.012 | | -0.083 | | 0.148 | | -0.047 | | -0.101 | |  |  |
| Tanzania | TZA | 3.355 | 2.040 | 0.407 | 0.908 | 0.608 | 0.121 | 0.271 | 0.262 | 0.543 | | 0.011 | | -0.291 | | 0.232 | | -0.024 | | -0.208 | |  |  |
| Yemen | YEM | 3.166 | 1.825 | 0.350 | 0.991 | 0.576 | 0.111 | 0.313 | -0.072 | -0.073 | | 0.014 | | -0.013 | | -0.020 | | 0.017 | | 0.004 | |  |  |

**Appendix 11.** Conservation indices for each impact category by country

| **Country** | **ISO3** | **Climate** | **Marine** | **Land** |
| --- | --- | --- | --- | --- |
| Australia | AUS | 0.331 | 0.629 | 0.624 |
| Bangladesh | BGD | 0.622 | 0.211 | 0.344 |
| Bahamas | BHS | 0.270 | 0.270 | 0.304 |
| Brazil | BRA | 0.205 | 0.610 | 0.324 |
| Cuba | CUB | 0.312 | 0.532 | 0.433 |
| Djibouti | DJI | 0.513 | 0.004 | 0.088 |
| Dominican Republic | DOM | 0.357 | 0.513 | 0.588 |
| Egypt | EGY | 0.339 | 0.599 | 0.183 |
| Eritrea | ERI | 0.370 | 0.235 | 0.213 |
| Fiji | FJI | 0.643 | 0.393 | 0.190 |
| Haiti | HTI | 0.363 | 0.224 | 0.249 |
| Indonesia | IDN | 0.479 | 0.636 | 0.618 |
| India | IND | 0.467 | 0.418 | 0.276 |
| Kenya | KEN | 0.204 | 0.581 | 0.263 |
| Sri Lanka | LKA | 0.334 | 0.197 | 0.705 |
| Myanmar (Burma) | MMR | 0.332 | 0.090 | 0.128 |
| Malaysia | MYS | 0.218 | 0.444 | 0.317 |
| Philippines | PHL | 0.332 | 0.530 | 0.470 |
| Papua New Guinea | PNG | 0.417 | 0.182 | 0.098 |
| French Polynesia | PYF | 0.312 | 0.468 | 0.000 |
| Saudi Arabia | SAU | 0.278 | 0.116 | 0.104 |
| Sudan | SDN | 0.190 | 0.396 | 0.128 |
| Singapore | SGP | 0.388 | 0.238 | 0.237 |
| Solomon Islands | SLB | 0.567 | 0.127 | 0.067 |
| Somalia | SOM | 0.254 | 0.137 | 0.170 |
| Timor-Leste | TLS | 0.317 | 0.289 | 0.063 |
| Tanzania | TZA | 0.278 | 0.675 | 0.512 |
| Yemen | YEM | 0.129 | 0.171 | 0.176 |

**Appendix 12.** Outlier countries for A) mean pressure and B) conservation index for each pressure category.


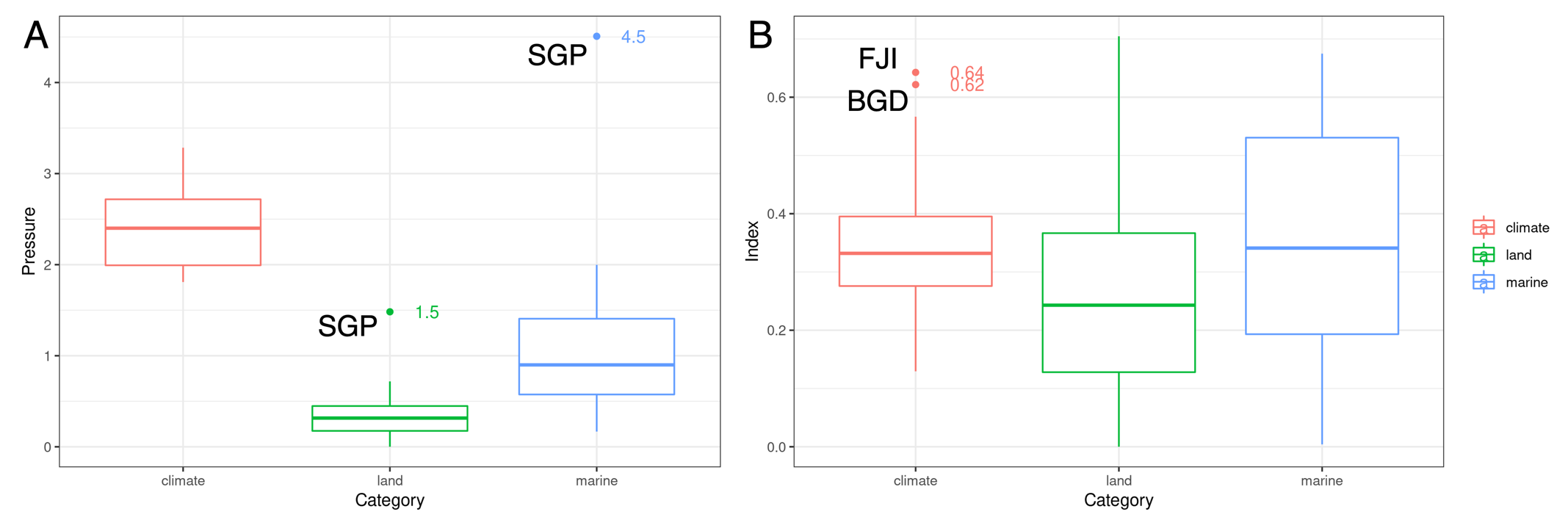


**Appendix 13.** Data distribution of the individual pressures of the cumulative human impact metric by pressures driver category across A) bioclimatic units within countries and B) bioclimatic units.


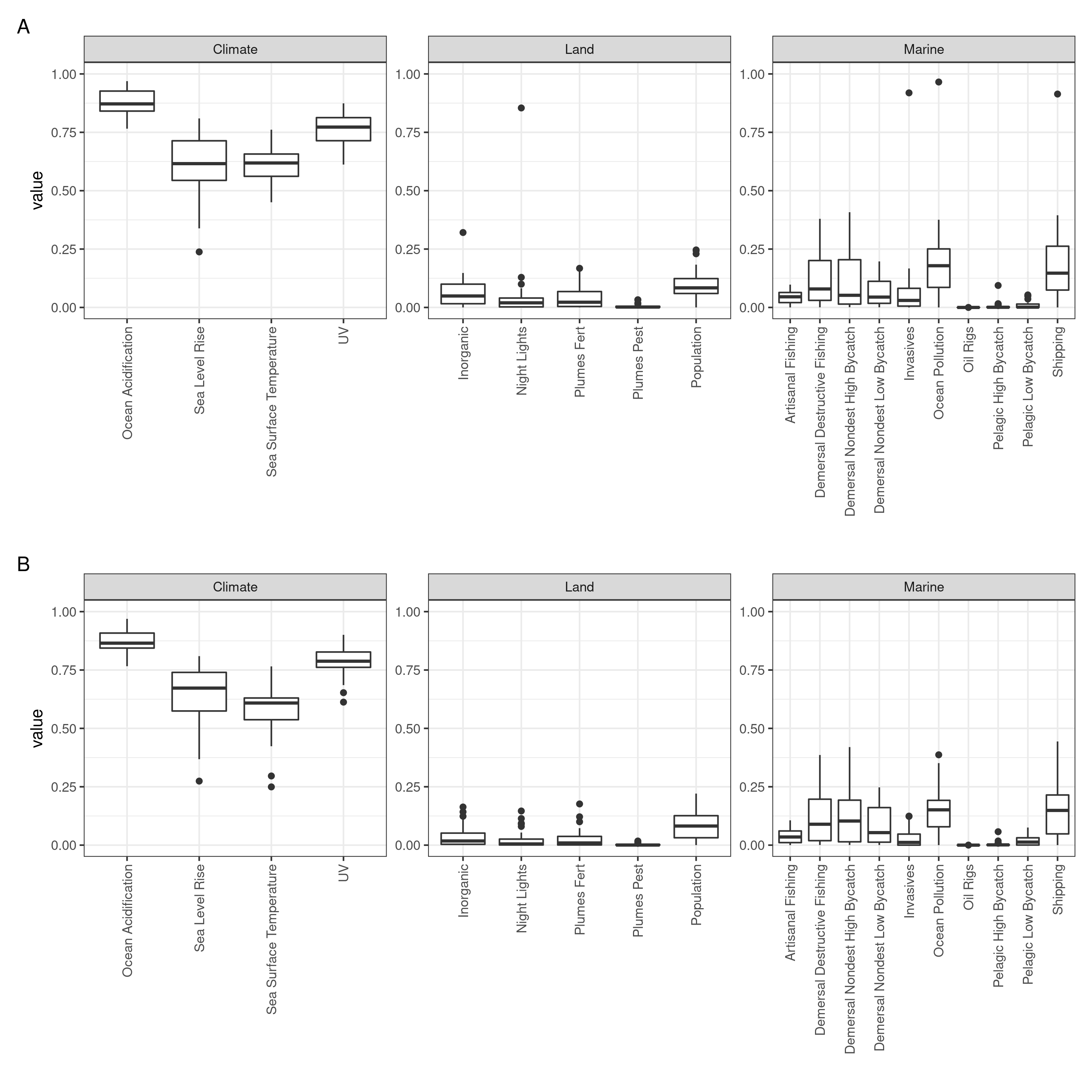


**References**

Ayalon, I., Rosenberg, Y., Benichou, J.I.C., Campos, C.L.D., Sayco, S.L.G., Nada, M.A.L., Baquiran, J.I.P., Ligson, C.A., Avisar, D., Conaco, C., Kuechly, H.U., Kyba, C.C.M., Cabaitan, P.C. & Levy, O. (2021). Coral Gametogenesis Collapse under Artificial Light Pollution. *Current Biology*, 31, 413-419.e3.

Beyer, H.L., Kennedy, E.V., Beger, M., Chen, C.A., Cinner, J.E., Darling, E.S., Eakin, C.M., Gates, R.D., Heron, S.F., Knowlton, N., Obura, D.O., Palumbi, S.R., Possingham, H.P., Puotinen, M., Runting, R.K., Skirving, W.J., Spalding, M., Wilson, K.A., Wood, S., Veron, J.E. & Hoegh‐Guldberg, O. (2018). Risk‐sensitive planning for conserving coral reefs under rapid climate change. *CONSERVATION LETTERS*, 11, e12587.

Boström-Einarsson, L., Babcock, R.C., Bayraktarov, E., Ceccarelli, D., Cook, N., Ferse, S.C.A., Hancock, B., Harrison, P., Hein, M., Shaver, E., Smith, A., Suggett, D., Stewart-Sinclair, P.J., Vardi, T. & McLeod, I.M. (2020). Coral restoration – A systematic review of current methods, successes, failures and future directions. *PLoS ONE*, 15, e0226631.

Burke, L., Reytar, K., Spalding, M. & Perry, A. (2011). *Reefs at Risk Revisited*. World Resources Institute.

Cabré, M.M., Gallagher, K.P. & Li, Z. (2018). Renewable Energy: The Trillion Dollar Opportunity for Chinese Overseas Investment. *China & World Economy*, 26, 27–49.

Chan, N.C.S. & Connolly, S.R. (2013). Sensitivity of coral calcification to ocean acidification: a meta-analysis. *Glob Change Biol*, 19, 282–290.

Chen, C., Hellmann, J., Coffee, J., Murillo, M. & Chawla, N. (2015). University of Notre Dame Global Adaptation Index.

Crippa, M., Guizzardi, D., Muntean, M., Olivier, J.G.J., Schaaf, E., Solazzo, E., Vignati, E., European Commission, & Joint Research Centre. (2018). *Fossil CO2 emissions of all world countries: 2018 report.*

Dudley, N. (2008). *Guidelines for applying protected area management categories*. IUCN.

Fabricius, K.E. (2005). Effects of terrestrial runoff on the ecology of corals and coral reefs: review and synthesis. *Marine Pollution Bulletin*, 50, 125–146.

Giffin, A.L., Brown, C.J., Nalau, J., Mackey, B.G. & Connolly, R.M. (2020). Marine and coastal ecosystem-based adaptation in Asia and Oceania: review of approaches and integration with marine spatial planning. *Pac. Conserv. Biol.*, 27, 104–117.

Gill, D.A., Mascia, M.B., Ahmadia, G.N., Glew, L., Lester, S.E., Barnes, M., Craigie, I., Darling, E.S., Free, C.M., Geldmann, J., Holst, S., Jensen, O.P., White, A.T., Basurto, X., Coad, L., Gates, R.D., Guannel, G., Mumby, P.J., Thomas, H., Whitmee, S., Woodley, S. & Fox, H.E. (2017). Capacity shortfalls hinder the performance of marine protected areas globally. *Nature*, 543, 665–669.

Guinotte, J.M., Buddemeier, R.W. & Kleypas, J.A. (2003). Future coral reef habitat marginality: temporal and spatial effects of climate change in the Pacific basin. *Coral Reefs*, 22, 551–558.

Hagger, V., Worthington, T., Saunders, M., Amano, T., Landis, E., Zganjar, C., Wilson, K., Mumby, P., O’Brien, K., Friess, D., Brown, B., Adame, F., Morrison, T. & Lovelock, C. (in prep). Socio-economic drivers of global mangrove conservation success.

Halpern, B.S., Frazier, M., Potapenko, J., Casey, K.S., Koenig, K., Longo, C., Lowndes, J.S., Rockwood, R.C., Selig, E.R., Selkoe, K.A. & Walbridge, S. (2015). Spatial and temporal changes in cumulative human impacts on the world’s ocean. *Nat Commun*, 6, 7615.

Hanson, J.O., Rhodes, J.R., Butchart, S.H.M., Buchanan, G.M., Rondinini, C., Ficetola, G.F. & Fuller, R.A. (2020). Global conservation of species’ niches. *Nature*, 580, 232–234.

Heron, S.F., Maynard, J.A., van Hooidonk, R. & Eakin, C.M. (2016). Warming Trends and Bleaching Stress of the World’s Coral Reefs 1985–2012. *Sci Rep*, 6, 38402.

Hughes, T.P., Kerry, J.T., Álvarez-Noriega, M., Álvarez-Romero, J.G., Anderson, K.D., Baird, A.H., Babcock, R.C., Beger, M., Bellwood, D.R., Berkelmans, R., Bridge, T.C., Butler, I.R., Byrne, M., Cantin, N.E., Comeau, S., Connolly, S.R., Cumming, G.S., Dalton, S.J., Diaz-Pulido, G., Eakin, C.M., Figueira, W.F., Gilmour, J.P., Harrison, H.B., Heron, S.F., Hoey, A.S., Hobbs, J.-P.A., Hoogenboom, M.O., Kennedy, E.V., Kuo, C., Lough, J.M., Lowe, R.J., Liu, G., McCulloch, M.T., Malcolm, H.A., McWilliam, M.J., Pandolfi, J.M., Pears, R.J., Pratchett, M.S., Schoepf, V., Simpson, T., Skirving, W.J., Sommer, B., Torda, G., Wachenfeld, D.R., Willis, B.L. & Wilson, S.K. (2017). Global warming and recurrent mass bleaching of corals. *Nature*, 543, 373–377.

ICRI. (2020). Members.

IPCC. (2019). *2019 Refinement to the 2006 IPCC Guidelines for Naitonal Greenhouse Gas Inventories*.

IUCN & UNEP-WCMC. (2019). *The World Database on Protected Areas (WDPA)*.

Jennings, S. & Polunin, N.V. (1996). Impacts of Fishing on Tropical Reef Ecosystems. *Ambio*, 25, 44–49.

Macreadie, P.I., Fowler, A.M. & Booth, D.J. (2011). Rigs-to-reefs: will the deep sea benefit from artificial habitat? *Frontiers in Ecology and the Environment*, 9, 455–461.

Mora, C., Myers, R.A., Coll, M., Libralato, S., Pitcher, T.J., Sumaila, R.U., Zeller, D., Watson, R., Gaston, K.J. & Worm, B. (2009). Management Effectiveness of the World’s Marine Fisheries. *PLoS Biol*, 7, e1000131.

OECD, European Union, & Joint Research Centre - European Commission. (2008). *Handbook on Constructing Composite Indicators: Methodology and User Guide*. OECD.

Perry, C.T., Alvarez-Filip, L., Graham, N.A.J., Mumby, P.J., Wilson, S.K., Kench, P.S., Manzello, D.P., Morgan, K.M., Slangen, A.B.A., Thomson, D.P., Januchowski-Hartley, F., Smithers, S.G., Steneck, R.S., Carlton, R., Edinger, E.N., Enochs, I.C., Estrada-Saldívar, N., Haywood, M.D.E., Kolodziej, G., Murphy, G.N., Pérez-Cervantes, E., Suchley, A., Valentino, L., Boenish, R., Wilson, M. & Macdonald, C. (2018). Loss of coral reef growth capacity to track future increases in sea level. *Nature*, 558, 396–400.

Shick, J.M., Lesser, M.P. & Jokiel, P.L. (1996). Effects of ultraviolet radiation on corals and other coral reef organisms. *Global Change Biol*, 2, 527–545.

Sorensen, J. (2000). Baseline 2000 Background Report: The Status of Integrated Coastal Management as an International Practice (Second Iteration), 168.

UN. (2015). *Sendai Framework for Disaster Risk Reduction 2015-2030*.

UNEP. (2018). *Analysis of international funding for the sustainable management of coral reefs and associated ecosystems*.

UNEP-WCMC, WRI & TNC. (2018). *Global distribution of coral reefs, compiled from multiple sources including the Millennium Coral Reef Mapping Project*. updated by UNEP-WCMC, Includes contributions from IMaRSUSF and IRD, . - IMaRS-USF (2005) and Spalding et al. (2001). Cambridge (UK): UNEP World Conservation Monitoring Centre.

Vega Thurber, R.L., Burkepile, D.E., Fuchs, C., Shantz, A.A., McMinds, R. & Zaneveld, J.R. (2014). Chronic nutrient enrichment increases prevalence and severity of coral disease and bleaching. *Glob Change Biol*, 20, 544–554.

Waldron, A., Mooers, A.O., Miller, D.C., Nibbelink, N., Redding, D., Kuhn, T.S., Roberts, J.T. & Gittleman, J.L. (2013). Targeting global conservation funding to limit immediate biodiversity declines. *Proceedings of the National Academy of Sciences*, 110, 12144–12148.

Wilson, K.L., Tittensor, D.P., Worm, B. & Lotze, H.K. (2020). Incorporating climate change adaptation into marine protected area planning. *Glob Change Biol*, 26, 3251–3267.

World Bank. (2020). *Doing Business 2020: Comparing Business Regulation in 190 Economies*. Washington, DC: World Bank.

Wurtsbaugh, W.A., Paerl, H.W. & Dodds, W.K. (2019). Nutrients, eutrophication and harmful algal blooms along the freshwater to marine continuum. *WIREs Water*, 6.
